# Strategies to Enhance Logic Modeling-Based Cell Line-Specific Drug Synergy Prediction

**DOI:** 10.3389/fphys.2020.00862

**Published:** 2020-07-28

**Authors:** Barbara Niederdorfer, Vasundra Touré, Miguel Vazquez, Liv Thommesen, Martin Kuiper, Astrid Lægreid, Åsmund Flobak

**Affiliations:** ^1^Department of Clinical and Molecular Medicine, Norwegian University of Science and Technology, Trondheim, Norway; ^2^Department of Biology, Norwegian University of Science and Technology, Trondheim, Norway; ^3^Barcelona Supercomputing Center, Barcelona, Spain; ^4^Department of Biomedical Laboratory Science, Norwegian University of Science and Technology, Trondheim, Norway; ^5^The Cancer Clinic, St. Olav’s University Hospital, Trondheim, Norway

**Keywords:** logic modeling, synergy prediction, high-influence nodes, cell signaling network, drug combination

## Abstract

Discrete dynamical modeling shows promise in prioritizing drug combinations for screening efforts by reducing the experimental workload inherent to the vast numbers of possible drug combinations. We have investigated approaches to predict combination responses across different cancer cell lines using logic models generated from one generic prior-knowledge network representing 144 nodes covering major cancer signaling pathways. Cell-line specific models were configured to agree with baseline activity data from each unperturbed cell line. Testing against experimental data demonstrated a high number of true positive and true negative predictions, including also cell-specific responses. We demonstrate the possible enhancement of predictive capability of models by curation of literature knowledge further detailing subtle biologically founded signaling mechanisms in the model topology. *In silico* model analysis pinpointed a subset of network nodes highly influencing model predictions. Our results indicate that the performance of logic models can be improved by focusing on high-influence node protein activity data for model configuration and that these nodes accommodate high information flow in the regulatory network.

## Introduction

Drug combinations are anticipated to advance cancer therapy by targeting multiple trajectories of the complex signaling crosstalk regulating cancer cell fate (Al-Lazikani et al., 2012; Hyman et al., 2017; Senft et al., 2017). Drug combinations may act synergistically and postpone the emergence of resistance. Empirical testing of limited numbers of drug combinations has already led to the discovery of several proven and promising drug combinations. These include the well-established use of MEK inhibitors in combination with BRAF inhibitors in melanoma patients (e.g., trametinib and dabrafenib) (Larkin et al., 2014; Robert et al., 2015; Long et al., 2016), or cyclin-dependent kinase 4/6 inhibitors (e.g., palbociclib) in combination with anti-estrogen therapy in breast cancer (Cristofanilli et al., 2016). However, due to the vast number of possible drug combinations, it is not feasible to efficiently and economically test the combinatorial drug space exhaustively. For example, in the Broad Institute’s Drug Repurposing Hub (Corsello et al., 2017) 128 compounds are currently annotated in the disease area oncology. Testing these compounds in pairs would result in 8128 combinations. This combinatorial explosion poses a bottleneck to the discovery of novel synergistic drug combinations.

In recent years, logic models have been successfully applied to predict drug responses and to prioritize drug combinations (Klinger et al., 2013; Miller et al., 2013; Flobak et al., 2015; Vitali et al., 2016; Eduati et al., 2017; Silverbush et al., 2017) including a recent application of patient-specific models (Eduati et al., 2020). These models build on a vast amount of signaling knowledge that has been uncovered over the years and archived in several databases like KEGG (Kanehisa and Goto, 2000), Reactome (Fabregat et al., 2018), or SIGNOR (Perfetto et al., 2016; Licata et al., 2019). However, annotated interactions suffer from study bias. For well-studied proteins often a multitude of functions have been described and many interactions have been annotated. Consequently, these proteins may appear as hubs, while interactions for other proteins are less well documented (Invergo and Beltrao, 2018). The use of perturbation data for model training (Klinger et al., 2013; Miller et al., 2013; Eduati et al., 2017; Silverbush et al., 2017) overcomes some of the challenges related to gaps in signaling knowledge by addition or removal of signaling influences as well as providing context specificity. However, such experiments are time-consuming and substantially increase the experimental load. Additionally, explorative perturbation studies are difficult to realize in a clinical setting. Thus, approaches enabling the use of logic modeling without this kind of training data might help us employ modeling approaches for pre-clinical and clinical applications at a later stage.

Few studies, excluding strictly statistical models, have investigated the use of baseline molecular data for cell-specific model calibration, e.g., molecular data like gene-expression solely from an unperturbed system (Flobak et al., 2015; Silverbush et al., 2017; Béal et al., 2019). Silverbush et al. (2017) used their model, initially informed with perturbation data to a specific cell line, to predict combinations for another cell line using only mutation data, validating the majority of the predicted combinations. In a recent DREAM challenge (Menden et al., 2019), participating teams used different machine-learning techniques to predict combination effects for 910 drug combinations using baseline molecular data across multiple cancer cell lines from different origins. In contrast, Jaeger et al. (2017) have explored the use of a generic signaling network without incorporating any cell-specific activity data. They reported a strong correlation between molecular features, such as mutations status and molecular subtype, and synergy strength in their validation data set, suggesting that predictive models can be generated without depending on perturbation data for model training.

A challenge in generating these models using only baseline data arises from the difficulty to correctly assess activity profiles for cell line components. It has been previously shown that a combination of different data types is beneficial in predicting drug responses (Iorio et al., 2016). While genomic and transcriptomic data are often abundantly available, proteomics and in particular phosphoproteomics data is much less available, although it might allow a more direct assessment of protein activity. As the challenge of understanding and translating these data into activity states for logic model calibration remains, only a limited amount of signaling components can be accurately assessed for their activity, thus calling for explorations that can provide guidelines as to which proteins should be probed for model calibration.

For a useful and ready application of logic modeling in a pre-clinical and clinical setting, it is necessary to enable high-quality predictions for a wider range of systems, such as complete cell line panels. In this study, we therefore set out to investigate the use of a single signaling knowledge network to predict synergistic drug combinations for four cancer cell lines derived from gastric, colorectal or prostate cancer, by calibrating the general model to cell-specific models using their baseline activity data. In addition, we investigated how specific model features impacted the predictive power of these models. Predictions were tested against our recently performed drug combination screen data (Flobak et al., 2019). Our results show that model calibration with a protein activity profile combining information from literature-curated and omics-inferred data increases predictive sensitivity. Network refinements based on literature knowledge accounting for subtle biologically founded mechanisms improve the model’s predictive performance. Further, *in silico* exploration indicates that nodes with high betweenness centrality and closeness centrality, and whose removal reduces network efficiency, have a higher influence on predictions. This can provide a prioritized list of signaling entities whose activity must be correctly assessed for model calibration.

## Materials and Methods

### Testing Data

Drug combination data for model testing was obtained from our previously performed high-throughput screening (Flobak et al., 2019). The following cancer cell lines were the focus of this study: AGS (gastric adenocarcinoma), COLO 205 (colorectal cancer), DU-145 (prostate cancer), and SW-620 (colorectal cancer). Drug combination effects were classified as synergistic or non-synergistic according to Highest Single Agent model (HSA). The HSA model assumes synergy if the effect of a combination is greater than that achieved by any of the single drugs alone (Berenbaum, 1989). For this, the HSA excess was calculated as *Viability(Drug A* + *Drug B) − min[Viability(Drug A*, *Drug B)]*. Combinations with mean HSA excess across doses of ≤−0.11 were classified as synergistic. Among the 19 drugs tested in all combinations, we observed that the SF inhibitor (targeting PTEN) was involved in the majority of observed synergies. Due to further lack of good characterization of this inhibitor for off-target effects against other kinases, we excluded it from further study. Hence, the testing set included 153 pairwise drug combinations from 18 inhibitors (Table 1).

**TABLE 1:** List of inhibitors.

Colloquial name	Abbreviation	Inhibitor	Primary HGNC target(s)	Node in network	PubChem CID
MAP3K7i	5Z	(5Z)-7-oxozeaenol (LL-Z1640-2)	MAP3K7	MAP3K7	9863776
AKTi	AK	Akt Inhibitor VIII (AKTi-1,2)	AKT1, AKT2, AKT3	AKT_f	135398501
MAPK14i	BI	Doramapimod (BIRB0796)	MAPK14	MAPK14	156422
GSK3i	CT	CHIR 99021 (CT99021)	GSK3A, GSK3B	GSK3_f	9956119
MEKi	PD	PD0325901	MAP2K1, MAP2K2	MEK_f	9826528
PI3Ki	PI	PI-103	PIK3CA, PIK3CB, PIK3CD	PIK3CA	9884685
CTNNB1i	PK	Toxoflavin (PKF118-310)	CTNNB1	CTNNB1	66541
JNKi	JN	JNK-IN-8 (JNK Inhibitor XVI)	MAPK8, MAPK9, MAPK10	JNK_f	57340686
RSKi	D1	BI-D1870	RPS6KA1, RPS6KA3, RPS6KA2, RPS6KA6	RSK_f	25023738
IKBKBi	60	BI605906 (BIX02514)	IKBKB	IKBKB	23652660
JAKi	RU	Ruxolitinib (INCB18424)	JAK1, JAK2	JAK_f	25126798
TGFBRi	SB	SB-505124	TGFBR1, ACVR1B, ACVR1C	TGFBR1, ACVR1C	56924523
CK1i	D4	D4476	CSNK1D, TGFBR1	CK1_f, TGFBR1	6419753
MYCi	F4	10058-F4	MYC	MYC	1271002
STAT3i	ST	Static	STAT3	STAT3	2779853
PDPK1i	G2	GSK2334470	PDPK1	PDPK1	46215815
ROCK1i	G4	GSK 429286	ROCK1	ROCK1	11373846
SYKi	P5	PRT 062607 (P505-15)	SYK	SYK	44462758

*Colloquial name indicates names for inhibitors used throughout the paper to improve the readability of the article. For computational analysis, inhibitors were identified using the indicated abbreviation.*

### Construction of Regulatory Network – CASCADE 2.0

**FIGURE 1:**
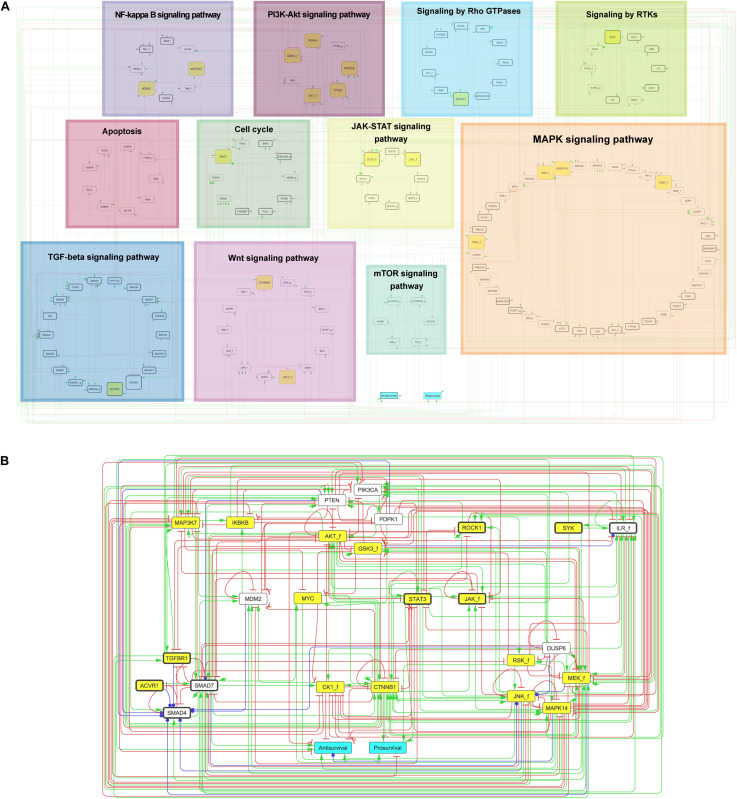
Prior knowledge network – CASCADE 2.0. Drug targets are highlighted in yellow boxes. Model outputs are indicated in turquoise. Nodes are linked by directed and signed interactions: green arrows denote activation; red arrows denote inhibition; blue arrows denote dual interaction. **(A)** Complete PKN. Nodes are categorized by primary pathways determined by KEGG (Kanehisa and Goto, 2000) and Reactome (Fabregat et al., 2018) (see section “Materials and Methods” for details). **(B)** Reduced PKN showing topology of generic logical model reduced by GINsim model reduction for drug target and output nodes. Nodes representing a family, a complex of proteins or a gene are annotated with “_f”, “_c,” and “_g”, respectively. Nodes additional to previously published PKN (Flobak et al., 2015) are indicated with thick black borders.

The published prior knowledge network (PKN) for the adenocarcinoma cancer cell line AGS (Flobak et al., 2015) was manually extended using SIGNOR (Perfetto et al., 2016), KEGG (Kanehisa and Goto, 2000), and relevant recent scientific publications (PubMed), to cover the 18 proteins targeted by small-molecule inhibitors (Table 1). Specifically, the KEGG database (Kanehisa and Goto, 2000) was used to extract proteins relevant to generic signaling pathways. Focusing on drug targets, the PKN was extended with signaling knowledge obtained from scientific literature. Each protein was either named after its official gene symbol or with its official genesymbol_f (i.e., AKT_f) if several isoforms are represented by a single node in the model (“f” stands for family); with its official genesymbol_g if the node represents a gene; or with symbol_c if the node represents a protein complex. Nodes informative of cellular phenotypes related to cell growth were identified and linked to the two output nodes “Prosurvival” and “Antisurvival.” This enabled the quantification of the effect of drug treatment simulations. All network and model constructions were done using GINsim (Naldi et al., 2009). Annotated regulatory interactions can be found in cascade_2.0.tsv at https://github.com/druglogics/cascade. Figure 1 was generated using Cytoscape 3.7.1 (Shannon et al., 2003) by grouping nodes by primary pathways from KEGG (Kanehisa and Goto, 2000), via DAVID analysis (Huang et al., 2009a, b), and Reactome (Fabregat et al., 2018). Nodes were associated with manually defined consensus pathways from these resources.

### Model Calibration and Simulation

The nodes in the regulatory network are connected by signed and directed interactions, representing activating or inhibiting regulatory events. Logical formalism was used to describe the regulatory rules of the network, defining the activity of each node by its upstream regulator using the Boolean operators *AND*, *OR*, and *NOT*, respectively, represented with &, |, and ! in the notation of the rules. Generally, all activating or inactivating regulators of nodes were linked by *OR*, while activators and inactivating regulators were combined by *AND NOT*. Exceptions from these rules were made for complexes or proteins acting in combination to activate a downstream node (Supplementary Table 2). All nodes except for the two phenotypic output nodes *Prosurvival* and *Antisurvival* can occupy the two Boolean states 1, describing an active node, or 0, describing an inactive node. The two output nodes were assigned the value multi-level values 0, 1, 2, or 3 (Supplementary Table 1). We note that after model refinement also the node FOXO_f was multivalued.

Additionally, the following generic rule had to be adjusted to compute a stable state and stop state oscillation in the model:

MAPK14=MAP2K3|MAP2K4|!DUSP1

This means that MAPK14 is more likely to be active. Specifically, MAPK14 will be active in the model if:

(1) MAP2K3 and/or MAP2K4 are active despite active DUSP1.
(2) MAP2K3 and MAP2K4 are inactive and DUSP1 is inactive.

For the generation of cell line-specific models, we next collected molecular activity information for the four cell lines addressed in the present study: the gastric adenocarcinoma AGS, the colorectal COLO 205 and SW-620, and the prostate DU-145 cancer cell lines. For each cell line, we generated three different sets of baseline activity data.

(1) Omics-inferred activity profile: using the pathway activity inference algorithm PARADIGM (Vaske et al., 2010), protein activity was inferred from cell line copy number variations and gene expression data obtained from the Cancer Cell Line Encyclopedia (Barretina et al., 2012).
(2) Literature informed activity profile: PKN node protein activity was collected from protein activity observations described in the scientific literature.
(3) Combined activity profile: since different sources for protein activity data may complement each other, and even do not always agree (Costello et al., 2014; Iorio et al., 2016), we generated a third set of baseline activity data by integrating the omics-inferred and literature-derived observations, where we prioritized literature-derived over omics-inferred activity states.

Cell-specific models were generated by modifying the general Boolean rules until nodes in the stable state of the model represented the given activity profile. First, nodes that were inactive in the stable state but supposed to be active according to the baseline data were subjected to this rule change to increase their chance of being active. In the first round, this only applied to nodes for which also the respective negative regulators of the node were active according to the baseline data. In the second round, negative regulators of nodes that were active in the computed stable state but supposed to be inactive were subjected to this rule change to inactivate the node of interest. These steps were repeated until the stable state of the model agreed with the baseline activity data. Thus, the resulting cell line models were derived from the same PKN but constituted by different logic rules. Modifications were done following the principle of Occam’s razor, where few changes were prioritized over many changes. For details regarding baseline activity data, model calibration and modified rules, see Supplementary Text S1 and Supplementary Tables 4–11.

Simulation of the model was performed with the software GINsim (Version 2.9.4) (Naldi et al., 2009), as described in Flobak et al. (2015). Drug perturbations were simulated by fixing the activity of nodes representative of drug targets to 0 and observing changes to the steady state behavior of the model. The outcome of single and double perturbations in the model simulations was assessed by measuring their effects on *Viability*, where *Viability* was calculated by subtracting the simulated output value of the *Antisurvival* output node from the output value of the *Prosurvival* node. A combination (double perturbation) that decreased *Viability* more than each single drug alone was classified as synergistic.

To enable simulations of perturbations when no stable state could be reached, the model was reduced using the model reduction algorithm provided by GINsim (Naldi et al., 2009), retaining the nodes targeted by our drugs of interest, the two output nodes, and nodes involved in self-loops that could not be removed. The initial states of the remaining nodes were first set to zero. A hierarchical transition graph was computed to analyze the effect of the perturbation on the phenotypic output nodes.

To evaluate model performance, we additionally computed the chance of obtaining the predicted number of True Positives (TP), False Positives (FP), True Negative (TN), and False Negatives (FN) by randomly selecting the predicted number of synergies per model and comparing it to the validation set (*N* = 100,000).

### Baseline Activity Data

A literature-derived baseline activity profile was collected for SW-620, AGS, COLO 205, and DU-145 cells by reviewing published articles by searching in PubMed for [Node] [Cell line], as in Flobak et al. (2015). For the AGS cell line, we reused the previously collected protein activity described in Flobak et al. (2015). The tool PARADIGM was used to infer the state of proteins from copy number variations and gene expression data from Cancer Cell Line Encyclopedia (degree = 7) (Vaske et al., 2010; Barretina et al., 2012), generating omics-inferred activity profiles. The combined activity profile was built by overlaying the omics-inferred activity state with the literature-derived activity state, giving priority to literature-derived data whenever in conflict with the omics-inferred state.

### Evaluation of Model Performance

Model-generated predictions were classified as TP, FP, TN and FN, by comparing the predicted synergies to the observed synergies in our drug screen (Flobak et al., 2019). To evaluate model performance, we have calculated a list of parameters. Matthews’s correlation coefficient (MCC) is a metric giving an overall impression on model performance accounting for unbalanced data (Chicco, 2017). Sensitivity was calculated as *TP/(TP* + *FN)* evaluating how many of the observed synergies we can predict. The positive predictive value calculated as *TP/(TP + FP)* evaluates how many of the predicted synergies are TPs. The negative predictive value calculated as *TN/(TN + FN)* evaluating how many of the combinations not predicted to be synergistic are TNs.

### Model Refinement

Model refinement was guided by biological insights that potentially underly false negative predictions. For this, the literature was searched for biological mechanisms regarding investigated drug combinations and for molecular mechanisms of single drugs that could explain single drug effects. We investigated reported signaling crosstalk between pairs of proteins targeted by drugs observed to act synergistically but lacking model synergy prediction. We focused on strategies relating to “shortest path” connections and logic rules. A summary of performed changes is listed below, while an extensive description can be found in the Supplementary Text S1.

(1) FOXO_f regulation and activity.
This was motivated by biological insights that could underlie the false negative prediction of MAPK37 inhibitor (5Z) with the PIK3CA inhibitor (PI) in the AGS cell line model.
  (a) The logic rule defining FOXO_f was adapted to cover an activity of 0, 1, or 2, depending on whether one or both regulators, NLK or AKT_f, is active or inactive.
  (b) The edge from CK1 to FOXO_f was removed, giving priority to the kinases that have been reported to distinctly regulate FOXO1’s nuclear exclusion (Kim et al., 2010; Mendes-Pereira et al., 2012; Monsalve et al., 2015).

(2) PDPK1 and PIK3CA signaling in PKN.
This was motivated by false negative prediction of the PDPK1 inhibitor (G2) with the PI3KCA inhibitor (PI) in the AGS and SW-620 cell line model.
  (a) PDPK1 is now positively regulated by RTPK_f, representing active PDPK1 when growth signals are present and independent of active PIK3CA (Mora et al., 2004; Anjum and Blenis, 2008).
  (b) Joint regulation of AKT_f by PDPK1 and PIK3CA is now represented by the inclusion of a new node PIP3, jointly activated by PDPK1 and PIK3CA and inhibited by PTEN. The logic rule for AKT_f was changed to [(mTORC2_c | ILK) & PIP3] & !PPP1CA.
  (c) To represent reduced potency of the G2 inhibitor (Knight, 2011; Najafov et al., 2011) in the presence of high PIK3CA signaling, the rule for PIP3 was further modified for cell lines with high PIK3CA inferred from single drug viability data.
  (d) The logical rule for S6K_f was modified to reflect effective downregulation upon exposure to the G2 inhibitor.
  (e) RSK_f is now active upon the presence of both PDPK1 and ERK_f (Anjum and Blenis, 2008).

(3) mTORC1/S6K and MYC signaling in PKN.
This was done to more directly link mTORC1/S6K signaling to the *Prosurvival* output.
  (a) A new node MXD1 was included, negatively regulated by both RSK_f and S6K_f, negatively regulating *Prosurvival* via inhibition of MYC (Zhu et al., 2008).

### Evaluation of High-Influence Nodes

The identification of high-influence nodes in the four cell lines models has been studied by sequentially perturbing (i.e., creating a mutation for) each node whose activity is then either fixed (measured activity in model) or inverted (1 – measured activity in model). In each case, this generates a mutated model that is subsequently analyzed: additional mutations are applied to each mutated model, corresponding to single drug target perturbation or a combination of two drug targets perturbation, resulting in 2^∗^(fixed model | inverted model) ^∗^ 144^∗^ (nodes) ^∗^ 171 (drug combinations) mutated models to test for each cell line. Using the bioLQM library (Naldi, 2018), stable states are computed for each mutated model and results are compared with the WT model (non-mutated model), for which stable states have also been computed. Similar to the simulation method used before (see section “Model Calibration and Simulation”), each mutated model for a particular drug combination was assessed with a synergy prediction (TP, TN, FP, and FN). The influential character of a node is assessed by monitoring whether the fixation or the inversion of its activity changed the synergy predictions compared to the WT analysis. By quantifying the sum of changes in synergy predictions, e.g., number of TP gains, number of TN gains, we rank each node in each cell line: the higher the number, the more changes occurred in the mutated model, providing a measure for node influence. All files are available in https://github.com/druglogics/influential-nodes.

### Identification of High-Influence Node Features

To determine node features that are characteristic of high-influence nodes, we used them in a Random Forest classifier (randomForest R package version 4.6-14). This classifier provided the Gini importance metric, which measures how useful each feature is in making the classification into high and low influence nodes. We conducted a batch of experiments considering separately the different types of features (numeric, pathways, etc.), whether the nodes were of high-importance in a particular cell line, in all cell lines, or in any cell line. The analysis was run with both balanced and unbalanced data in terms of nodes in each of the two classes. Similar results were obtained both with unbalanced and balanced data. The code for this batch analyses can be found in https://github.com/druglogics/influential-nodes/tree/master/taskNodeAssessment/results/randomForest.

### Analysis of High-Influence Nodes to Investigate Putative Synergy Mechanisms

Potential drug synergy mechanisms were investigated in subgraphs containing high-influence nodes for drug combinations of interest. For this, nodes whose inversion or fixation lead to a complex attractor were excluded from the analysis. In contrast to a stable state where all nodes remain fixed in their activity, a complex attractor is defined by all or some nodes oscillating in their activity (Zañudo and Albert, 2013). After obtaining a combination- and cell line-specific list of high influential nodes, a subgraph of the complete network was generated to obtain the perturbated pathway structures. Next, the furthest downstream nodes of the subgraph (nodes without outgoing edges) were used to obtain nodes whose activity is altered by additional fixation or inversion of those influential nodes upon simulation of drug combination effects. These nodes were added to the subgraph structure, while all sink nodes, excluding output nodes, were removed. The analysis was performed using R version 3.5.3 and tidyr (0.8.3), dplyr (0.8.1) and igraph (1.2.4.1).

## Results

### Calibration of a Comprehensive PKN With Information From Literature and Omics Data Enables Drug Synergy Predictions

We set out to build a logic model that can be used to predict drug combination responses. Specifically, the model was designed to enable predictions for 153 drug combination responses of 18 drugs obtained in our recent cancer cell line screen (see Flobak et al., 2019 and Materials and Methods). With this strategy, the final model predictions for each drug combination could be tested against observations from our previous *in vitro* study to assess prediction quality. In brief, to represent the signaling crosstalk between proteins targeted by each single drug, we expanded our previously published PKN developed for predicting the response to seven drugs and their combinations in the AGS gastric adenocarcinoma cell line (Flobak et al., 2015) to represent or increase signaling coverage for the additional 11 drug targets. The constructed PKN (Figure 1) represents a comprehensive signaling network of 144 nodes and 366 interactions. The expansions (Figure 1A, nodes with thick black borders) include entire pathways (TGF beta-, JAK- STAT-, and Rho GTPase-) and substantial augmentation of several other pathways.

To enable logic rule fine-tuning and calibration to cancer cell line-specific models, the PKN was first translated into a generic Boolean model capturing the dynamics of the network. Cell line-specific models were generated by calibrating the PKN to each of the three sets of protein activity data (see “Materials and Methods” section for details).

(1) Omics-inferred activity profile: this approach yielded activity state suggestions for the majority of nodes for all cell lines.
(2) Literature informed activity profile: this enabled estimation of activity states of 21, 9, 41, and 26 nodes for the AGS, COLO 205, DU-145, and SW-620 cells, respectively.
(3) Combined activity profile: this approach yielded activity state suggestions for the majority of nodes for all cell lines.

To assess the capacity of our models to correctly predict cell line-specific synergistic drug combinations, we compared simulated cell survival to observed outcomes in our large experimental screen (Flobak et al., 2019) (see section “Materials and Methods” for details). We focused our analysis on the effect of the different protein activity data sets on model performance for two of the cell lines: AGS and SW-620. Overall, we found that combining literature-derived and omics-inferred activity profiles produced models with a higher (AGS) or the same (SW-620) number of true positive predictions, compared to models informed with the omics-inferred activity profile, and fewer false positives than literature-informed models (see Supplementary Text S1 and Supplementary Figures 2, 3). These models were thus better suited for guiding preclinical screening efforts since fewer false negative synergy predictions resulted, while no true positive predictions were lost. If the experimental screen had omitted to test all drug combinations predicted to be ineffective, the number of combinations for AGS cells could have been reduced from 153 to 10, while still discovering 5 of the 15 observed synergistic drug pairs.

### Network and Model Refinement Improve Predictions of Efficient Drug Combinations

**FIGURE 2:**
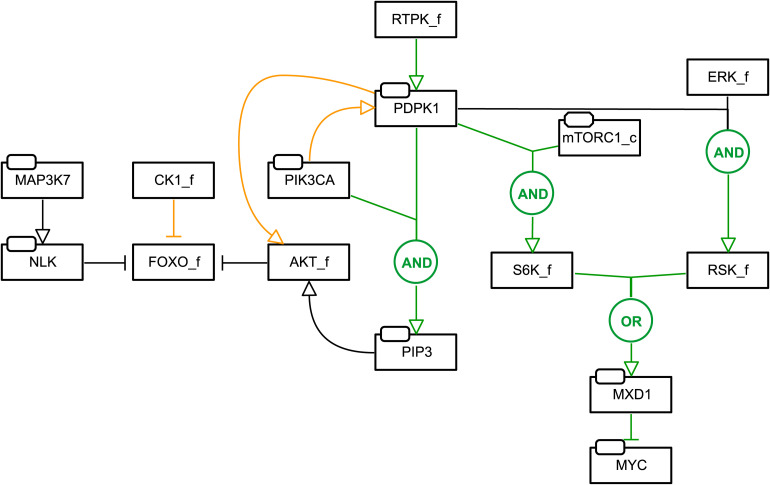
Network and model refinements. Added edges are indicated in green, while removed edges are indicated in orange. For new edges or changes to Boolean rules, the Boolean operator describing the activity of a node is indicated in circles. Nodes representing family or a complex of proteins are indicated by “_f” and “_c”, respectively. For example: FOXO_f regulation and activity was modified by changing the logic rule defining FOXO_f to cover an activity of 0, 1, or 2, depending on whether one or both regulators, NLK or AKT_f, is active or inactive, and by modifying the topology with the edge from CK1 to FOXO_f removed, giving priority to the kinases that have been reported to distinctly regulate FOXO1’s nuclear exclusion (Kim et al., 2010; Mendes-Pereira et al., 2012; Monsalve et al., 2015). See Section “Materials and Methods” for a full description of these changes.

When prioritizing drug combinations for screening, ideally one would not want to miss possibly effective combinations by having false negatives. One possible explanation for the low sensitivity from our initial set of models is that causal interactions covered in existing knowledge databases, e.g., SIGNOR (Perfetto et al., 2016; Licata et al., 2019) and KEGG (Kanehisa and Goto, 2000), are incomplete and lack context under which different interactions occur. Since signaling knowledge is likely to be more exhaustively represented in scientific literature than in databases, we started a series of refinements to increase the predictive power of our models. For this we adjusted the signaling network and logic rules informed by careful investigation of signaling information in scientific literature, aiming at better recapitulating the underlying biological mechanisms for the studied cell lines. A total of seven network modifications were made. A summary of changes is shown in Figure 2 (see section “Materials and Methods” for a more comprehensive description and Supplementary Text S1 for details).

**FIGURE 3:**
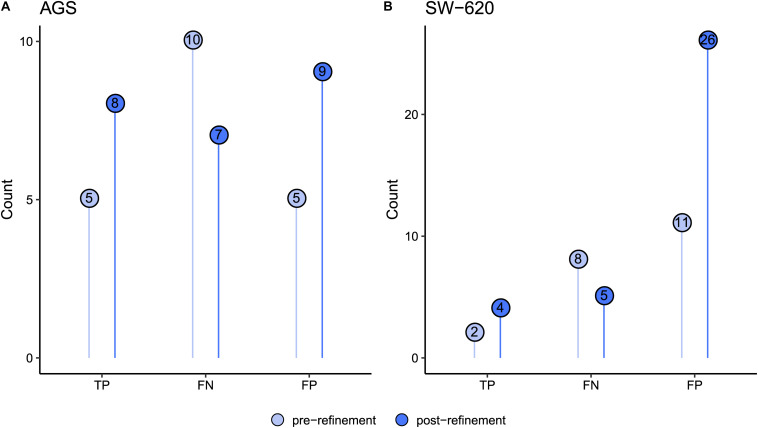
Comparison of predictions pre- and post-refinement of the PKN and model using the combined activity profile. Figures show prediction improvements after refinement for **(A)** AGS and **(B)** SW-620 model. Numbers in dots indicate observations for true positives (TP), false negatives (FN), and false positives (FP).

To evaluate whether the model refinements resulted in improved performance, we generated AGS- and SW-620-calibrated versions of the refined model and performed new drug simulations. As illustrated in Figure 3, the refined model generated more true positive predictions for AGS and SW-620 cells compared to the pre-refinement models. For example, drug combinations involving the MAP3K7 inhibitor could now correctly be predicted to be synergistic in AGS cells. The SW-620 model now predicted additional synergistic combinations involving the PI3Ki. Overall, the decrease in false negative predictions makes the model more suitable for economizing preclinical screening efforts.

To investigate the performance of our model for other cancer cell lines, we performed simulations for two additional cell lines: COLO 205 and DU-145. For this, cell-specific models were calibrated to the combined baseline protein activity profiles. Comparing the overall performance of the four cell line-specific models (Table 2), we observed that the AGS cell line model showed highest sensitivity of observed synergies. All models showed improved positive predictive value compared to random prediction performance, indicating an increase in truly synergistic combinations among predictions compared to random guessing (Table 2). Low performance of the COLO 205 model could be attributed to the scarcity of literature-reported protein activity data or the prior network not optimally representing all relevant signaling mechanisms involved in the tested drug responses in this cell line. Prediction performance in our study, with an overall balanced accuracy of 0.64 and cell line-specific balanced accuracy ranging from 0.73 to 0.51 for our best and worst performing model, is comparable to the balanced accuracy achieved in the recent DREAM challenge, with an overall average balanced accuracy of 0.63, and the best performing individual team obtaining a balanced accuracy of 0.69 (Menden et al., 2019).

**TABLE 2:** Synergy predictions for AGS, SW-620, COLO 205, and DU-145 cells using the refined PKN.

Refined model	AGS		SW-620		COLO 205		DU-145	
	Prediction	Random	Prediction	Random	Prediction	Random	Prediction	Random
TP	8	1.8	4	1.8	1	0.8	3	1.6
FN	7	13.3	5	7.2	7	7.2	14	15.4
TN	128	121.7	117	114.8	131	130.8	125	123.6
FP	9	15.3	26	28.2	14	14.2	11	12.4
Sensitivity [%]	53.3	11.2	44.4	19.8	12.5	9.8	17.6	9.1
PPV [%]	47.1	9.9	13.3	5.9	6.7	5.2	21.4	11.1
NPV [%]	94.8	90.1	95.9	94.1	94.9	94.8	90.0	88.9
MCC	0.44	0.00	0.16	0.00	0.02	0.00	0.10	0.00

*Models were informed using combined activity data. TP, true positive; FN, false negative; TN, true negative; FP, false positive; PPV, positive predictive value; NPV, negative predictive value; MCC, Matthews’s correlation coefficient. Random **–** expected average count for respective values by chance when selecting the number of predicted synergistic combinations from the pool of possible drug combinations (n = 100,000).*

### Model Predictions Enable Reduction in Experimental Load

**FIGURE 4:**
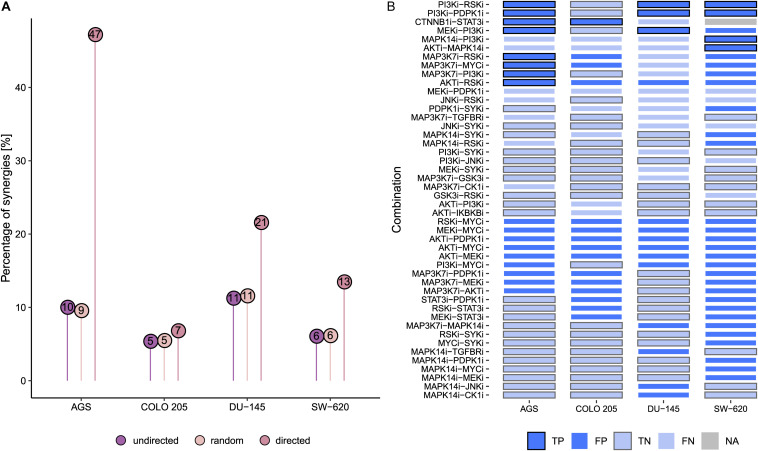
Predictive performance of cell-specific models. **(A)** Percentage of synergies across all tested combinations (undirected, *n* = 153) vs. combinations predicted to be synergistic (directed, *n*_AGS_ = 17, *n*_COLO 205_ = 15, *n*_DU–145_ = 14, *n*_SW–620_ = 30). **(B)** Predictions across different cell line models. True negative predictions across all cell lines are not shown (*n* = 107). True predictions are indicated by thick frame borders (black frames for true positives, and gray frames for true negatives). Abbreviations: true positive (TP), false positive (FP), true negative (TN), and false negative (FN). Color fill: light blue – negative predictions, dark blue – positive predictions, gray – NA.

Next, we tested how our model predictions fared against all experimentally characterized drug combinations (Flobak et al., 2019). Of 153 possible pairwise drug combinations tested, on average 8% of combinations were observed to be synergistic in the four cell lines. In contrast, a screen of only the drug combinations predicted to be effective by our current models would have increased this drug screen efficiency to 47% for AGS, and efficiencies for DU-145, SW-620, and COLO 205 would have been 21%, 13%, and 7%, respectively (Figure 4A). In other words, for three of the cell lines, experimental testing of drug combination effects guided by logic model predictions would have substantially reduced the need for an exhaustive drug screen to discover a high percentage of synergies. Overall, we find that the number of combinations to be experimentally tested could have been reduced to 12% (76 of 612 combinations + cell lines) and thereby increasing the detection rate of synergies 2.6-fold, from 8 to 21%.

To gain more insight into cell line-specific drug combination predictions, we systematically compared predictions across the four cell lines as well as their agreement with the drug combination response. As illustrated in Figure 4B, of 34 combinations predicted to be synergistic by at least one of the cell-specific models, six are predicted by all models and nine combinations are predicted for three of the tested cell lines. Specifically, the model correctly identifies the joint targeting of the PI3Ki and PDPK1i as synergistic in AGS, DU-145, and SW-620 cells and non-synergistic in COLO 205. Of note, several of true positive synergy predictions involve drug targets jointly targeting the PI3K-AKT and MAPK-signaling pathway such as combined targeting of RSK and PI3K. Of the observed synergistic drug combinations, 12 are never predicted by any of the cell models, indicating possible knowledge gaps. This includes any of the synergistic combinations involving JNKi and GSK3i. However, in several cases, synergies missed in one cell line are correctly predicted by other cell line models. This suggests that the underlying signaling graph encompasses the relevant interactions and components to cover these combination effects, while the cell model-specific logic rules might not always optimally represent the investigated cell line. This observation could result from the calibration data not containing the correct signaling activities, thus asking for node identification for which high-quality activity state data is highly desired.

### Identification of High-Influence Nodes Enables Improved Prediction Qualities

**FIGURE 5:**
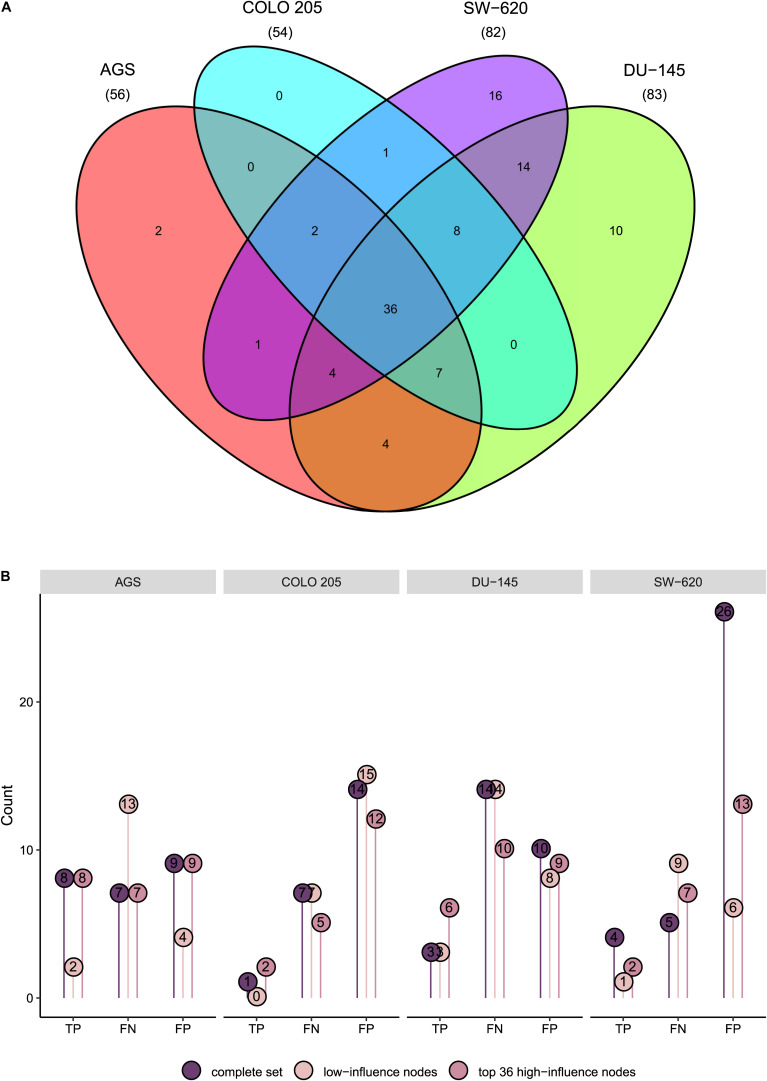
Cell-specific high-influence nodes. **(A)** Venn diagram displaying the number of distinct and shared influential nodes across cell lines. **(B)** True positive (TP), false negative (FN), and false positive (FP) predictions of cell line models using the complete set, the 38 nodes classified as non-influential in all cell lines or the top 36 high-influence nodes for each cell line of the combined baseline data for model calibration.

We next set out to examine the importance of correct node activity assessment, specifically posing the question of whether the correct assessment of activity for some nodes matter more than others. Inspired by previous publications exploring the identification of high-influence nodes (Gao et al., 2014; Puniya et al., 2016; Campbell et al., 2017; Pentzien et al., 2018; Rozum and Albert, 2018; Yang et al., 2018) as well as by the concept of target control in network biology, assuming that only a subset of nodes control the system (Gao et al., 2014; Yang et al., 2018), we pursued the goal to identify such nodes in our network as well as features that identify them. For each node we iteratively fixed its activity both at its stable state activity value and after activity inversion followed by computation of new synergy predictions. For each cell line model, we recorded all nodes whose inversion or fixation either changed any of the predictions or resulted in a system attractor where some nodes would cycle between activity and inactivity (i.e., in a complex attractor). Both node classes were designated to be high-influence nodes (see “Materials and Methods” section). Specifically, 56, 54, 83, and 82 nodes were classified as high influence nodes for the AGS, COLO 205, DU-145, and SW-620 model, respectively (Figure 5A). This corresponds to one to two thirds of all nodes in the model. The 36 high influence nodes shared by all cell lines include oncogenes KRAS, BRAF and the Wnt-signaling component CTNNB1. For 38 nodes, inversion or fixation did not affect predictions in any of the cell lines. Taken together, our results support the hypothesis that a subset of nodes may be most decisive for the state of the model (Gao et al., 2014; Puniya et al., 2016; Campbell et al., 2017; Pentzien et al., 2018; Rozum and Albert, 2018; Yang et al., 2018).

We next set out to investigate whether the use of baseline protein activity calibration data could be restricted to only high influential nodes. For this, we calibrated new models for each cell line using the combined baseline protein activity data for only the top 36 cell model-specific high-influence nodes. Of these, 8 high-influence nodes were shared among all cell lines, while 4, 2, and 12 nodes were distinct for AGS, COLO 205 and SW-620, respectively (Supplementary Figure 4). For comparison, we calibrated models for each cell line where we only used protein activity data for the 38 nodes identified as non-influential in all the cell line models (see Supplementary Text S1). Figure 5B shows predictions for these two new sets of models alongside predictions from the models calibrated to the complete baseline data. We find that models generally show equal or better performance when calibrated to high-influence nodes compared to either the complete set of baseline activity data or to low-influence nodes. Thus, our results indicate that acquisition of model calibration data can focus on a subset of nodes identified to be of high influence using a simulation approach as presented here.

To enable classification of nodes that are of high influence for the quality of model predictions prior to computationally exhaustive simulations, we investigated different node features. We annotated all nodes in the refined model for their structural network characteristics including betweenness centrality, out-degree and pathway cross-talk inhibition index (PCI), the latter quantifying the relative reduction of network efficiency (Latora and Marchiori, 2007; Jaeger et al., 2017). A complete list of analyzed network features is available in Supplementary Text S1. In addition, nodes were annotated with selected biological features encompassing association with KEGG-, Reactome-, and ACSN- pathways, Gene Ontology terms related to protein function, classification as drug targets related to the experimental screen used for testing model predictions, and for their classification as cancer census genes (tier 1), oncogene or tumor suppressor according to COSMIC (Sondka et al., 2018).

**FIGURE 6:**
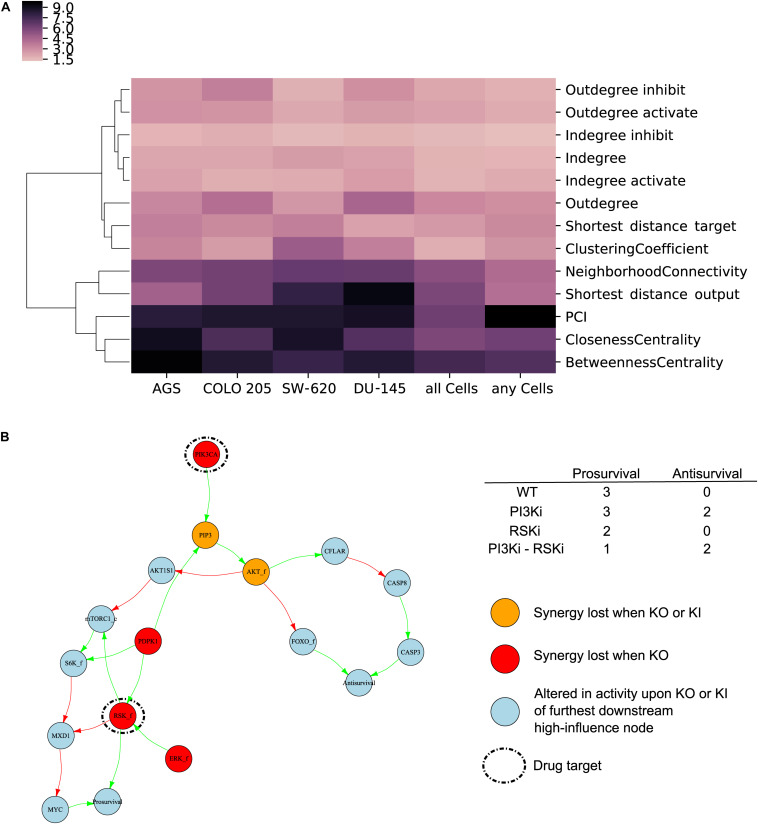
High-influence nodes are characterized by network-based features and may be used to propose synergy mechanisms. **(A)** Network-based feature importance. Gini importance for network features used by random forest to classify influential nodes in each cell lines, for all cell lines or of nodes influential in any cell lines. **(B)** Putative synergy mechanism of combined inhibition of the nodes PIK3CA and RSK_f in AGS cells. Simulation of combined application of PI3Ki and RSKi leads to a further decrease in “Prosurvival” compared to single drugs. Regulation of output nodes by the drug targets occurs both via mechanisms specific to each individual drug target as well as to downregulation of MYC only seen by joint inhibition of both drug targets. Color code: red – high-influence nodes whose fixation led to a loss in synergy prediction, yellow – high-influence nodes whose fixation and inversion led to a loss in synergy prediction, light blue – nodes whose activity was altered upon fixation or inversion of most downstream influential nodes in addition to simulation of perturbation of drug combination targets.

Using a random forest algorithm, we first tested if any of the network features could identify high-influence nodes. Cluster analysis of network feature Gini importance scores (Menze et al., 2009) showed that PCI, closeness centrality and betweenness centrality could be used to *a priori* identify high-influence nodes in a model (Figure 6A). Interestingly, these network properties provided better identification of high-influence nodes compared to annotated biological features (Supplementary Figure 5), indicating that emergent properties from network analyses complement traditional molecular biology knowledge. To further investigate the usability of the identified features, we produced scatter plots (Supplementary Figure 6) indicating that nodes with high influence in all or any cells tend to have higher PCI, betweenness centrality and closeness centrality compared to nodes with low influence. As PCI and betweenness centrality have a high and significant correlation (Pearson correlation of 0.90) either of these features could be used to identify high-influence nodes for a careful assessment of the baseline activity.

### High-Influence Nodes Can Propose Synergy Mechanisms

We next explored whether our approach for identifying influential nodes could additionally be used to elucidate potential synergy mechanisms. For this, we studied graph subsets around nodes of high-influence for drug combinations that were experimentally observed to be synergistic and correctly predicted by the model, as illustrated below for the example of PI3Ki in combination with RSKi in AGS cells. Nodes identified to be of high influence for a given drug synergy are in most instances not directly linked to the nodes depicting *Antisurvival* or *Prosurvival*, but rather impinge on them via multiple pathways that are involved in propagating the signal to the two phenotypic nodes. To investigate potential signal propagating mechanisms, we therefore further identified nodes whose activity was changed upon *in silico* experiments where we simultaneously inhibited the drug target nodes and in addition applied a knock-in or a knock-out to the furthest downstream nodes of the previously identified high-influential nodes. For the synergy of combined PIK3CA and RSK_f inhibition, a total of six nodes had been identified to be of high-influence (see red and yellow nodes in Figure 6B). The signal converges from the high influential nodes AKT_f and RSK_f. A total of 11 nodes (blue nodes in Figure 6B) including the two phenotypic outputs nodes were identified to be altered in their activity upon knock-in or knock-out of AKT_f and knock-out of RSK_f. Only for the high-influence nodes, knock-out or knock-in completely blocks the synergistic response, while inferences with the other nodes in the subgraph merely weakens the synergistic effect. Our model simulations indicated that inhibition of either PIK3CA and RSK_f independently alters the phenotypic output nodes “Antisurvival” and “Prosurvival” with a clear synergistic effect of inhibiting both (Figure 6B, upper right table). From the subgraph obtained from our *in silico* knock-out/knock-in experiments (Figure 6B) it can be seen that both PIK3CA and RSK_f indirectly regulate the node “MYC” by inhibition of its negative regulator MXD1. Based on these observations we hypothesize that this mechanism can be responsible for the synergistic decrease in “Prosurvival” achieved by combined targeting of PIK3CA and RSK_f. The here discussed example illustrates how our modeling approach for analyzing drug combination effects can be useful not only for prediction of drug synergies but also to propose putative synergy mechanisms.

## Discussion

Effective use of computational models to increase efficiency of preclinical drug screening needs system-specific models. We have investigated the feasibility of applying logic simulations of cell system models to obtain cell line-specific predictions of efficient drug combinations. We focused on simulation characteristics of high value for preclinical screening: the ability to discover all effective drug combinations, i.e., the quest to minimize false negative predictions, and the ability to identify combinations of interest even if they are synergistic in only a subset of cell lines. We show that our modeling approach can predict cell line-specific drug responses and present strategies to optimize logic modeling procedures by logic rule refinement and by identification of high-influence nodes for which accurate baseline protein activity data will likely be essential for obtaining well-performing models.

Optimal calibration of a model to protein activity states in a specific cell line is critical for a good predictive performance and is often achieved by using perturbation data (Klinger et al., 2013; Miller et al., 2013; Eduati et al., 2017; Silverbush et al., 2017). However, comprehensive perturbation data covering a wide range of drug combinations and biological systems are often not conveniently available for model calibration, even for pre-clinical systems, due to the cost of experiments. We therefore focus on strategies to calibrate cell-specific models with the use of baseline molecular data, a field which has up to now only been modestly studied (Flobak et al., 2015; Béal et al., 2019; Menden et al., 2019). We here demonstrate the generation of four cell line specific models from one PKN graph and further show that baseline activity profiles leveraging inference both from large scale omics data and high-quality small-scale data give the best possible ratio of true positive, false negative and false positive predictions. This is in line with observations from another group (Iorio et al., 2016), indicating that strategies to combine different types of calibration data, thereby reducing the uncertainty of estimated protein activity, are beneficial for model performance. The use of literature curated protein activity data in this study limits its scalability to a high number of different cell lines and its application in a clinical context. However, we expect that increasing availability of targeted phosphoproteomics data will allow for confident protein activity estimation and abrogate the constraint of manual activity curation for future investigations.

Construction of suitable PKNs poses a central challenge to the successful use of mechanistic models. PKN construction is hampered by the incompleteness of signaling database content compared to scientific literature, heavy bias toward much studied signaling mechanisms (Invergo and Beltrao, 2018) and lack of biological context that would allow a more specific design of PKNs to biological systems of interest, such as under which conditions, in which sequence and in which tissues causal interactions occur. Other studies indicate that such database shortcomings can to some extent be remedied by including the use of perturbation data for model training (Klinger et al., 2013; Miller et al., 2013; Eduati et al., 2017; Silverbush et al., 2017) or by performing iterative model refinement by comparison with experimental data and literature (Collombet et al., 2017). In this study, we succeeded in improving predictive performance of initial predictions by turning to the scientific literature and extracting additional knowledge for manual PKN refinement and logic rule adjustments. In a parallel study (Tsirvouli et al., 2019, submitted manuscript), we show how this PKN can be effectively extended and enhanced further by a middle-out approach leveraging the analysis of multi-omics data from the TCGA Colorectal Adenocarcinoma (COAD) cohort to guide the specification of the model for this cancer type.

Testing our logical model predictions for different cell lines from multiple origins against our comprehensive *in vitro* drug combination screen (Flobak et al., 2019) demonstrated that synergy predictions are enriched in experimentally observed synergies for three of the cell lines originating from gastric, colorectal and prostate tumors with a good ability to identify non-synergistic drug combinations (true negatives). Correctly predicted combinations include amongst other joint application of PI3Ki with PDPK1i or RSKi. The former combination has been previously reported to show synergistic effect in bladder cancer cells (Sathe et al., 2018) and is also observed to show a synergistic effect across multiple cancer cell line in our drug combination screen (Flobak et al., 2019). Since the states of roughly half (56%) of all network nodes with available baseline data were the same across cell lines, it can be expected that several predictions are shared among the different models. Interestingly, while taking the same PKN as starting point, models tailored to specific cell lines nevertheless displayed a high degree of cell line specificity. We observed that only roughly half of the combinations predicted to be synergistic were shared across three or more cell lines. These results further support the use of baseline data for model calibration applied in our previous study (Flobak et al., 2015) and show that this approach can be extended to multiple cell line models. Of interest for future study is the application of this approach to more advanced culture models such as organoids and xenograft models as well as prediction of a patient’s treatment response, potentially employing automated model generation and predictions to allow investigation of a larger number of drugs and experimental screening platforms.

Several different reference models have been developed to quantify synergism. Each of these models have different definitions and assumptions and as such they can show disagreement in synergy scoring (Vlot et al., 2019). In this study we have applied the HSA synergy metric to allow for the detection of as many as possible potentially synergistic drug combinations for pre-clinical screening. In order to binarized the drug combination data a synergy cut-off guided by literature and investigation of drug responses was applied, similar to other studies (Menden et al., 2019). When testing two alternative cut-offs that would result in approximately half or twice as high synergy calling compared to the one used in this study, we observe a generally increase in sensitivity with the more conservative experimental threshold, reflected by a decrease in FN prediction (Supplementary Figure 7 and Supplementary Table 27). This indicates that our model predictions were enriched in the strongest synergistic combinations. To enable a more fine-graded response of synergy strength, inclusion of additional multi-valued nodes or the use of a multi-valued logical model approach such as applied by Silverbush et al. (2017) may be investigated in future explorations.

Previous studies have indicated that a subset of nodes have the ability to affect the behavior of the overall network (Gao et al., 2014; Puniya et al., 2016; Campbell et al., 2017; Pentzien et al., 2018; Rozum and Albert, 2018; Yang et al., 2018). Translated to our example, this means that calibration of a set of nodes with such characteristics will determine the activity of the remaining nodes. In our efforts to identify such a subset of nodes, we specifically looked for nodes whose activity state affected prediction outcomes. The proportion of high-influence nodes among all 144 network nodes varied between cell lines from roughly half to one third. When cell-specific models were calibrated using only protein activity data for topmost high influential nodes, their performance was at least as good (increased or similar number of true positive and decrease in false positive predictions) as when using the complete baseline data set. It can be speculated that the improved functionality could be related to the fact that partially calibrated models have a high degree of freedom to find their optimal configuration. However, we show that the models calibrated to the lowest ranking quartile from the influential node assessment display a markedly poorer performance.

We also investigated whether high-influence nodes could be identified using network or biological features. Pentzien et al. (2018) proposed identification of high-influence nodes by assessing their determinative power, which quantifies the influence of a node over other nodes in a network taking both their degree as well as the Boolean logic into account (Heckel et al., 2013; Matache and Matache, 2016). They reported enrichment of essential genes among their high-influence nodes, while no commonalities of network features besides out-degree were found. We were unable to find biologically annotated function traits linked to high-influential nodes. However, we did observe that nodes with higher PCI, betweenness centrality and closeness centrality are overrepresented among the high-influence nodes. These findings indicate that network nodes associated with high information flow are good candidates to be included if an experimental assessment of protein activity is restricted to a subset of nodes in strategies seeking to increase effectiveness of predictive modeling. Strategies to identify high influential nodes, like the one we applied here, may further allow focus on a subset of nodes for an enrichment of the PKN with refined representations of network signaling mechanisms. Thus, our approach may alleviate two prominent bottlenecks: the limited capacity of current experimental methods for exhaustively determining protein activity states, e.g., through phosphoproteomics assays, and the burden to obtain accurate signaling network information for an entire PKN, including biological context of importance for the system to be modeled.

Mechanistic models may be explored to unravel a drug’s mechanism (Espinal-Enríquez et al., 2014) or to identify successful combination therapies by investigating multitarget drugs (Tang et al., 2013). In this study we focused on predicting the combination effect of a panel of small molecular inhibitors considering the inhibitors main annotated target. Polypharmacology is a well-known attribute of pharmacological compounds where unknown or unintended targets are commonly referred to as “off-target” effects. These unknown “off-target” effects pose significant challenges to the prediction of drug and drug combinations effects. While some drugs are highly specific, others may show a wide target profile. This also the case for the compounds investigated in this study (Flobak et al., 2019). A full exploration into predicting and testing drug effects on wider target profiles (i.e., including “off-targets”) is beyond the scope of this publication, but clearly an interesting path for future research.

In summary, we demonstrated the effective use of logic modeling to predict cell line-specific drug combination effects using baseline calibration data. Our findings show that both the underlying PKN and calibration data are critical for good predictions. Manual network refinement can be effective when several underlying molecular mechanisms are documented in scientific literature. In future investigations, it is also of interest to explore the curation of cell line-specific topologies from baseline data recapitulating the presence or absence of specific causal interactions in a specific cell line or context. While this is in theory possible from our approach, inferring the presence or absence of causal interactions from baseline data is not a trivial task. Our findings to restrict the assessment of stable state protein activity to high-influential nodes is a first step to economize the amount of calibration data that needs to be assessed with high confidence. These results suggest that improved curation of molecular interaction logical models, together with focused baseline calibration data sets can constitute the foundation for logic-model based drug synergy prediction for cancer cell lines in general.

## Data Availability Statement

All datasets generated for this study are included in the article/Supplementary Material or available under https://github.com/druglogics/influential-nodes.

## Author Contributions

BN, MK, AL, and ÅF contributed to conception and design of the study. BN, VT, and MV performed data curation and code development. BN and VT performed the formal analysis and led investigations of the study. BN, VT, and ÅF developed the methodology. MK, AL, and ÅF were responsible for project administration. LT, AL, and ÅF supervised the project. BN wrote the first draft of the manuscript. All authors contributed to manuscript revision, read and approved the submitted version.

## Conflict of Interest

The authors declare that the research was conducted in the absence of any commercial or financial relationships that could be construed as a potential conflict of interest.
