# Supplementary material for: Strategies to Enhance Logic Modeling-Based Cell Line-Specific Drug Synergy Prediction
Source: Front Physiol. 2020 Jul 28;11:862. doi: 10.3389/fphys.2020.00862 (PMC7399174; doi:10.3389/fphys.2020.00862)
Supplement: Supplementary file 7 [file Table_1.DOCX]

Supplementary Text

# Baseline activity data for model calibration

Three sets of calibration data were collected for each cell line: 1) omics-inferred activity data, 2) literature-curated activity data and 3) a combined activity profile of (1) and (2). For the AGS cell line, literature-curated activity data was reused from our previously published modelling effort with a reduced cut-off in needed references per protein activity (Flobak et al. 2015). In the combined activity profile, the literature-derived steady state was prioritized in case of conflict with the omics-inferred steady state. The combined activity profile held activity data different from omics-inferred activity profile suggestions for nine (31%), four (14%), eleven (27%) and seven (27%) proteins, for AGS, COLO 205, DU-145 and SW-620 cells, respectively (Supplementary Table 3).

We note that of the 129 proteins for which we could obtain baseline activity states across all cell lines, 63 varied in their activity among the four cell lines (Supplementary Figure 1A). This corresponds to 44% of all nodes in the model and includes proteins in the TGF-beta signaling pathway (e.g. SMAD1-7) and MAPK signaling pathways (e.g. JNK_f and MEK_f). We also observed drug response heterogeneity across the four cell lines. We find that, even though some combinations are effective in all four cell lines, each of them displays specific response profiles (Supplementary Figure 1B). These differences include combinations involving inhibitors targeting JNK, MEK1/2, MAP3K7 (TAK1) and PI3K. This means that while we can expect some across-model similarities in drug response predictions, we should also see several differences that, in the models, result from differences in observed protein activities.

# Model calibration

Cell line specific models were generated by adapting the logical rules in the generic Boolean model as described in the Material and Methods section, until the steady state of the model agreed with protein activity data for a specific cell line. Due to the substantial size of the model and the possibility that inferred protein activity states may contradict each other in the topology, it is inevitable that some inconsistencies may arise between the activity of nodes in the model and their reported activity in the calibration data. In addition, simulations are dependent on computable fixed points, i.e.: some Boolean rules must be adjusted so that the model can reach a stable state. Thus, in models calibrated towards the omics-inferred activity profile, 38 (29%) or 51 (39%) nodes could not be adjusted to reflect the baseline activity data for AGS and SW-620 cells, respectively. Here, the Paradigm scoring (S4 Data) was used to guide which nodes should be given priority. For models calibrated using the combined activity profile (omics-informed predictions augmented by literature-curated states), 37 (28%) and 44 (34%) nodes could not be adjusted to represent the curated steady state for AGS and SW-620 cells, respectively. In models calibrated toward the literature-derived activity profile, all the reported nodes’ activities complied with the reported activity data in the respective models, due to only a subset of nodes having a defined activity. Similar to the situation for calibration of AGS- and SW620 models, a fraction of the nodes could not be adjusted to reflect the annotated steady state for the later calibrated COLO 205 (36%) and DU-145 (30%) models to the combined activity data.

# Initial predictions of drug combination effects using different calibration data

For initial investigations we focused on two cancer cell lines: AGS and SW-620. For each of the three variant logic models calibrated to the three cell specific protein activity profiles (omics-inferred, literature-informed, combined), we simulated the effect of single and double small inhibitor treatment by fixing the nodes representing the protein targets of the inhibitors in the OFF state. For each simulation new stable states were computed and the ensuing change in the values of the output nodes Pro- and Anti-survival were recorded. In parallel, we generated randomly computed predictions, i.e., number of true positives, false positives, true negative and false negatives obtained when randomly selecting the number of predicted synergistic combinations by each of the models, to assess the quality of predictions.

The results, shown in Supplementary Figure 2A and B, show that models calibrated towards the activity profiles combining literature information and omics inference from large scale data performed best. Quantified prediction results can be found in Supplementary Table 12 and Table 13. Specifically, of the 15 experimentally observed synergies in AGS cells, predictions generated from models informed by the combined activity profile comprised five true positive with five false positive predictions. Models informed with the omics-inferred activity profile missed out on two synergistic combinations correctly predicted by models calibrated to the combined profile, however, with only one false positive combination prediction. The literature informed AGS model failed to predict one synergistic combination correctly predicted by the combined profile, with six of the predicted combinations being false positives (Supplementary Figure 2A). A similar trend was observed for SW-620 models with the model calibrated towards the literature-derived activity profile comprising the highest number of false positive predictions (Supplementary Figure 2B).

Comparing the performance of the models representing the two different cell lines, we found that AGS models showed a higher specificity compared to SW-620 models, where more than twice as many false positives are predicted. Nevertheless, even with calibration to the best performing combined baseline protein activity data, the AGS and SW-620 models missed out on ten and eight of the observed synergies, respectively. For example, no synergies were predicted when simulating combinations comprising the drug 5Z targeting MAP3K7 (TAK1), while 5Z is observed to be involved in a high number of synergistic combinations in AGS (see far left column in Supplementary Figure 3D). In the SW-620 model observed synergistic combinations involving the JN or PI inhibitors are never predicted to be synergistic (Supplementary Figure 3F). These findings confirm that a combination of sources for model calibration is beneficial, as also observed by others (Iorio et al. 2016). The predictive performance of the model further indicates that the PKN may not optimally represent all relevant interactions to describe the molecular mechanisms underlying the synergies observed in the drug screen.

# Network and model refinement

In the AGS cell line drug screen, we observed six drug combination synergies involving the MAP3K7i inhibitor targeting Transforming growth factor-beta-activated kinase TAK1 (MAP3K7). However, none of these were predicted by the model. The combination of the MAP3K7i and PI3Ki was also included in our previous work (Flobak et al. 2015). Given that our previous model was able to predict this synergistic combination, we investigated the current model in order to identify opportunities for improvement. In the experimental screen (Flobak et al. 2019), each of the inhibitors decreased cell viability when applied in isolation. However, in the AGS model simulations, only the inhibition of PI3KCA decreased the overall viability by increasing the activity of the *Antisurvival* node via activation of CASP8. Inhibition of MAP3K7 alone did not have an effect on any of the output nodes. A common node in the signalling paths from PIK3CA and MAP3K7 to *Antisurvival* is FOXO_f, additionally regulated by CK1_f. In the literature, it has been reported that AKT kinase isoforms negatively regulate the activity of several FOXO isoforms. The kinase CK1 has been described to further increase the negative regulation of FOXO1 upon its AKT-mediated phosphorylation on the residue S319 (Monsalve et al. 2015). Interestingly, TAK1-Nemo-like kinase (NLK) has been reported to negatively regulate FOXO1 independently of the PI3K/AKT signalling pathway (Kim et al. 2010). Additionally, the synthetic lethal inhibition of NLK in PTEN deficient cells has been described to be mediated by FOXO1 (Mendes-Pereira, Lord, and Ashworth 2012). Hence, we applied the following changes to the PKN:

- To address the reduced viability observed in the validation set by treatment with either the 5Z or PI inhibitor, the logical rule defining FOXO_f was adapted to cover an activity of 0, 1 or 2, depending on whether one or both regulators, NLK or AKT_f, are active or inactive. We note that this change is taking into account the single drug response data, thus the model is trained to single perturbation data.
- The edge from CK1 to FOXO_f was removed, giving priority to the kinases that have been reported to distinctly regulate FOXO1’s nuclear exclusion. This also reflects the synergy mechanism observed in our previous model (Flobak et al. 2015).

Another false negative prediction included the combinations of the PDPK1 inhibitor (PDPK1i) with the PIK3CA inhibitor (PI3Ki), observed to be synergistic in both the AGS and SW-620 cell line. In the model, PDPK1 is activated by PIK3CA, which further activates AKT. However, while it has been reported that the generation of PtdIns(3,4,5)P3 (PIP3) by PI3K is important in activating AKT due to co-localising AKT and PDPK1 to the plasma membrane, the PDPK1-dependent activation of RSK and S6K is independent of this (Mora et al. 2004; Anjum and Blenis 2008). Additionally, others described that the potency of the PDPK1i inhibitor varies among PDPK1’s different downstream targets. In contrast to the rapid inhibition of S6K phosphorylation by PDPK1, AKT phosphorylation was inhibited under moderate PI3K activity and RSK phosphorylation after 8 and 24 hours, respectively (Najafov et al. 2011; Knight 2011). This suggests that additional exposure of cells with PI3Ki and RSKi might further decrease viability of the cell lines by more potently inhibiting AKT and RSK. To reflect these observations, we applied the following changes:

- The rule for PDPK1 was changed so that this node will be active when RTPK_f is ON, representing active PDPK1 when growth signals are present independent of active PIK3CA. To continue reflecting the PIK3CA-independent activation of RSK and S6K by PDPK1 but joint activation of AKT_f, the PKN was adjusted to include a new node PIP3 positively regulated by the joint activity of PIK3CA and PDPK1, and inhibited by PTEN. The logical rule for AKT_f was changed to ((mTORC2_c | ILK) & PIP3) & !PPP1CA.
- The logical rule for RSK_f was adapted to represent its full activation when both PDPK1 and ERK_f are present.
- The logical rule for S6K_f was modified to reflect the downregulation upon exposure to the PDPK1i
- For cell lines whose viability decreased more than 20% upon exposure to the PI inhibitor, the logical rule for PIP3 was further modified. In those cases, the node is only regulated by PIK3CA considering the reduced potency of the G2 inhibitor (Najafov et al. 2011; Knight 2011). This adjustment was motivated by the hypothesis that cells susceptible to PI3K inhibition are likely to have an active PI3K-AKT pathway, including both for the AGS and SW-620 cell line.

Finally, we aimed to more directly link mTORC1/S6K signaling to the *Prosurvival* output, an important survival signaling pathway in cancer (Beauchamp and Platanias 2013). We found evidence that RSK_f and S6K_f inhibits MXD1, also known as Mad1, which is a protein competing with MAX for MYC and repressing its transcriptional activity (Zhu, Blenis, and Yuan 2008). This resulted into the following refinement:

- The node MXD1, negatively regulated by both RSK_f and S6K_f, was included in the model.

# High-influence nodes

To investigate possible benefits of focusing on the high influential nodes for model calibration, we calibrated new models for each cell line using only a two different subsets of the baseline protein activity data: (1) baseline activity data for top 36 cell line specific high-influence nodes and (2) baseline activity data for 38 nodes of low-influence in all cell lines. This means that, for these models, we did not use any baseline protein activity data for other nodes in the model. The baseline data were from the literature and omics-inferred combined set. The top 36 high-influence nodes were identified by counting the number of affected predictions for that respective cell line and using it for ranking the nodes (Supplementary Table 18). Also for these models not all nodes could be adjusted to represent the correct activity from the baseline data. For the AGS cell models eight out of 35 (23%) of the top 36 high-influence nodes with defined activity could not be adjusted for the high-influence model and nine of the 35 (26%) defined low-influence nodes could not be adjusted for the low-influence model. For the COLO 205 cell models 12 of 33 (36%) defined nodes of the top 36 high influence nodes could not be adjusted to represent correct stable state for the high-influence model and 15 of 33 (45%) defined low-influence nodes could not be adjusted for the low-influence model. For the DU-145 seven out of 35 (20%) defined nodes of the top 36 influential nodes could not be adjusted for the high-influence model and 12 out of 35 (34%) defined low-influence nodes could not be adjusted for the low-influence model. For the SW-620 cell line high-influence model two stable states were found with 11 and 10 out of 35 (31% and 29%) top high-influence nodes not agreeing with the SW-620 baseline activity data, respectively. For the low-influence node model ten out of 34 (30%) defined low-influence nodes could not be adjusted. Prediction results are shown in Supplementary Table 14-18. Tables summarizing the adjusted rules for the different models are summarized in Supplementary Table 19-26.

To investigate a priori identification of nodes from topological features, the following numerical node features were collected for each node:

1. Closeness Centrality (Cytoscape V 3.7.1)
2. Betweenness Centrality (Cytoscape V 3.7.1)
3. Indegree (Cytoscape V 3.7.1)
4. Outdegree (Cytoscape V 3.7.1)
5. Neighborhood Connectivity (Cytoscape V 3.7.1)
6. Shortest distance target (node --> target, R 3.5.1, dodgr)
7. Shortest distance output (node --> target, R 3.5.1, dodgr)
8. Pathway Cross-talk Inhibition index (PCI) (Jaeger et al. 2017; Latora and Marchiori 2007)
9. Outdegree inhibit (R 3.5.1)
10. Outdegree activate (R 3.5.1)
11. Indegree inhibit (R 3.5.1)
12. Indegree activate (R 3.5.1)
13. Clustering Coefficient (Cytoscape V 3.7.1)

# References

Anjum, Rana, and John Blenis. 2008. “The RSK Family of Kinases: Emerging Roles in Cellular Signalling.” *Nature Reviews. Molecular Cell Biology* 9 (10): 747–58. https://doi.org/10.1038/nrm2509.

Beauchamp, E. M., and L. C. Platanias. 2013. “The Evolution of the TOR Pathway and Its Role in Cancer.” *Oncogene* 32 (34): 3923–32. https://doi.org/10.1038/onc.2012.567.

Flobak, Åsmund, Anaïs Baudot, Elisabeth Remy, Liv Thommesen, Denis Thieffry, Martin Kuiper, and Astrid Lægreid. 2015. “Discovery of Drug Synergies in Gastric Cancer Cells Predicted by Logical Modeling.” *PLoS Computational Biology* 11 (8): e1004426. https://doi.org/10.1371/journal.pcbi.1004426.

Flobak, Åsmund, Barbara Niederdorfer, Vu To Nakstad, Liv Thommesen, Geir Klinkenberg, and Astrid Lægreid. 2019. “A High-Throughput Drug Combination Screen of Targeted Small Molecule Inhibitors in Cancer Cell Lines.” *Scientific Data* 6 (1): 237. https://doi.org/10.1038/s41597-019-0255-7.

Iorio, Francesco, Theo A. Knijnenburg, Daniel J. Vis, Graham R. Bignell, Michael P. Menden, Michael Schubert, Nanne Aben, et al. 2016. “A Landscape of Pharmacogenomic Interactions in Cancer.” *Cell* 166 (3): 740–54. https://doi.org/10.1016/j.cell.2016.06.017.

Jaeger, Samira, Ana Igea, Rodrigo Arroyo, Victor Alcalde, Begoña Canovas, Modesto Orozco, Angel R. Nebreda, and Patrick Aloy. 2017. “Quantification of Pathway Cross-Talk Reveals Novel Synergistic Drug Combinations for Breast Cancer.” *Cancer Research* 77 (2): 459–69. https://doi.org/10.1158/0008-5472.CAN-16-0097.

Kim, Sunhong, Yongsung Kim, Jiwoon Lee, and Jongkyeong Chung. 2010. “Regulation of FOXO1 by TAK1-Nemo-like Kinase Pathway.” *The Journal of Biological Chemistry* 285 (11): 8122–29. https://doi.org/10.1074/jbc.M110.101824.

Knight, Zachary A. 2011. “For a PDK1 Inhibitor, the Substrate Matters.” *Biochemical Journal* 433 (2): e1 LP-e2. http://www.biochemj.org/content/433/2/e1.abstract.

Latora, V., and M. Marchiori. 2007. “A Measure of Centrality Based on Network Efficiency.” *New Journal of Physics* 9 (6): 188–188. https://doi.org/10.1088/1367-2630/9/6/188.

Mendes-Pereira, Ana M., Christopher J. Lord, and Alan Ashworth. 2012. “NLK Is a Novel Therapeutic Target for PTEN Deficient Tumour Cells.” *PloS One* 7 (10): e47249. https://doi.org/10.1371/journal.pone.0047249.

Monsalve, Maria, Lars-Oliver Klotz, Ignacio Prieto-Arroyo, Cristina Sánchez-Ramos, Pavel Urbánek, Holger Steinbrenner, Ignacio Prieto-Arroyo, Pavel Urbánek, Holger Steinbrenner, and Maria Monsalve. 2015. “Redox Regulation of FoxO Transcription Factors.” *Redox Biology* 6 (December): 51–72. https://doi.org/10.1016/j.redox.2015.06.019.

Mora, Alfonso, David Komander, Daan M F van Aalten, and Dario R. Alessi. 2004. “PDK1, the Master Regulator of AGC Kinase Signal Transduction.” *Seminars in Cell & Developmental Biology* 15 (2): 161–70. https://doi.org/10.1016/j.semcdb.2003.12.022.

Najafov, Ayaz, Eeva M. Sommer, Jeffrey M. Axten, M. Phillip Deyoung, and Dario R. Alessi. 2011. “Characterization of GSK2334470, a Novel and Highly Specific Inhibitor of PDK1.” *The Biochemical Journal* 433 (2): 357–69. https://doi.org/10.1042/BJ20101732.

Zhu, Jidong, John Blenis, and Junying Yuan. 2008. “Activation of PI3K/Akt and MAPK Pathways Regulates Myc-Mediated Transcription by Phosphorylating and Promoting the Degradation of Mad1.” *Proceedings of the National Academy of Sciences of the United States of America* 105 (18): 6584–89. https://doi.org/10.1073/pnas.0802785105.

Supplementary Figures

[Supplementary Figure 1 - Differences in combined molecular activity for model calibration and synergy data for model testing. 7](#_Toc42120304)

[Supplementary Figure 2 - Comparison of predictions of PKN using different activity profiles. 8](#_Toc42120305)

[Supplementary Figure 3 – Predictions of AGS and SW-620 models using different activity profiles. 9](#_Toc42120306)

[Supplementary Figure 4 - Venn diagram of top 36 high-influential nodes per cell lines. 10](#_Toc42120307)

[Supplementary Figure 5 - Biological feature importance. 11](#_Toc42120308)

[Supplementary Figure 6 - Scatter plot for PCI, Betweenness centrality and Closeness centrality distributions of high- and low-importance nodes. 12](#_Toc42120309)

[Supplementary Figure 7 – Model predictions for different synergy cut-offs. 13](#_Toc42120310)


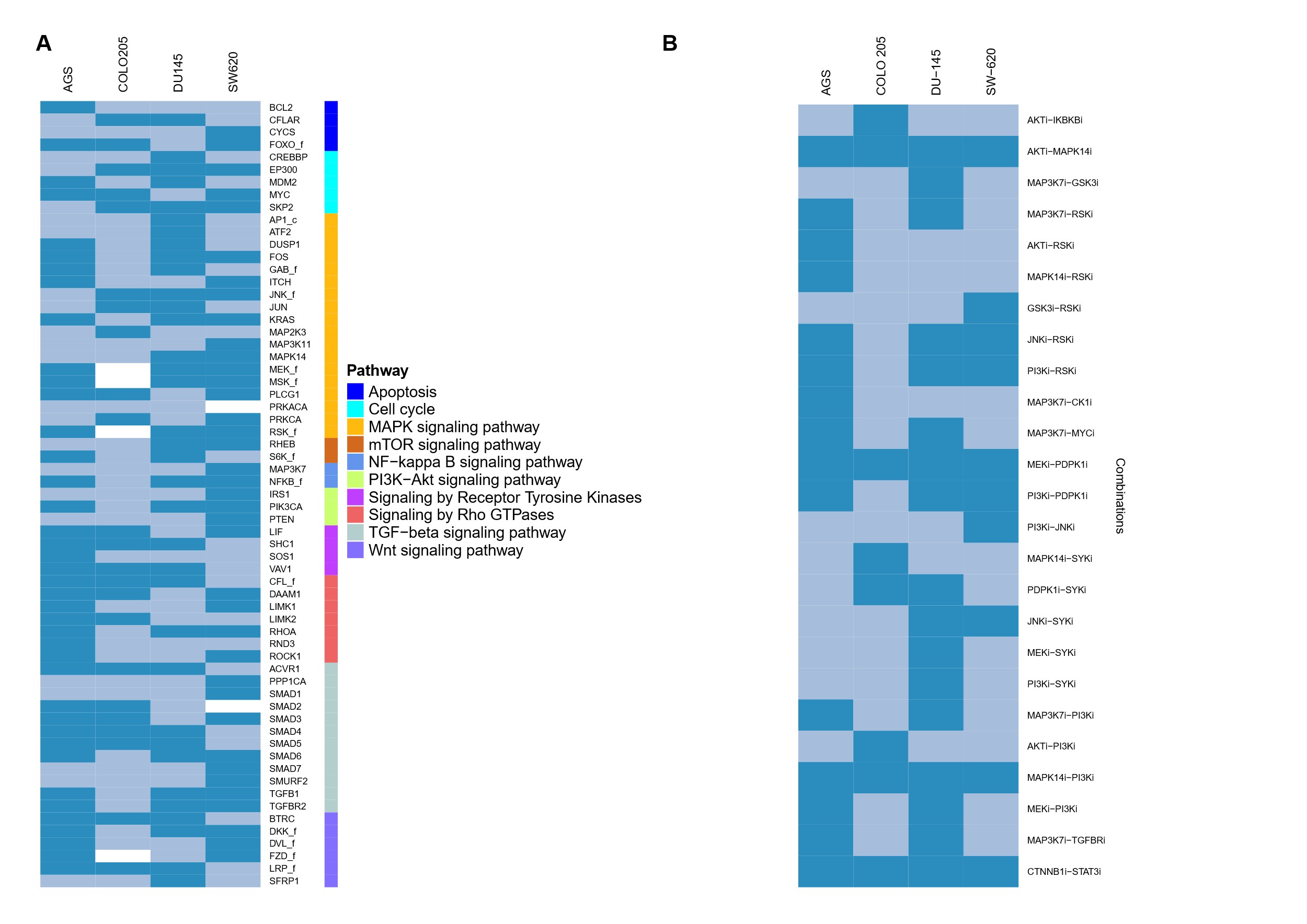


Supplementary Figure 1 - Differences in combined molecular activity for model calibration and synergy data for model testing.

(A) Heatmap of all 63 PKN nodes that vary in molecular activities in AGS, DU-145, SW-620 and COLO 205 cell lines in the combined activity profiles. Color scale: light blue – 0/inactive; dark blue – 1/active, white - NA. (B) Heatmap of all 25 synergies observed in at least one cell line excluding PTENi (SF). 21 combinations differ between AGS, DU-145, SW-620, COLO 205 cell lines according to Highest single agent. This represents 14% of all 153 combinations tested. Color scale: light blue – Non synergistic; dark blue – Synergistic


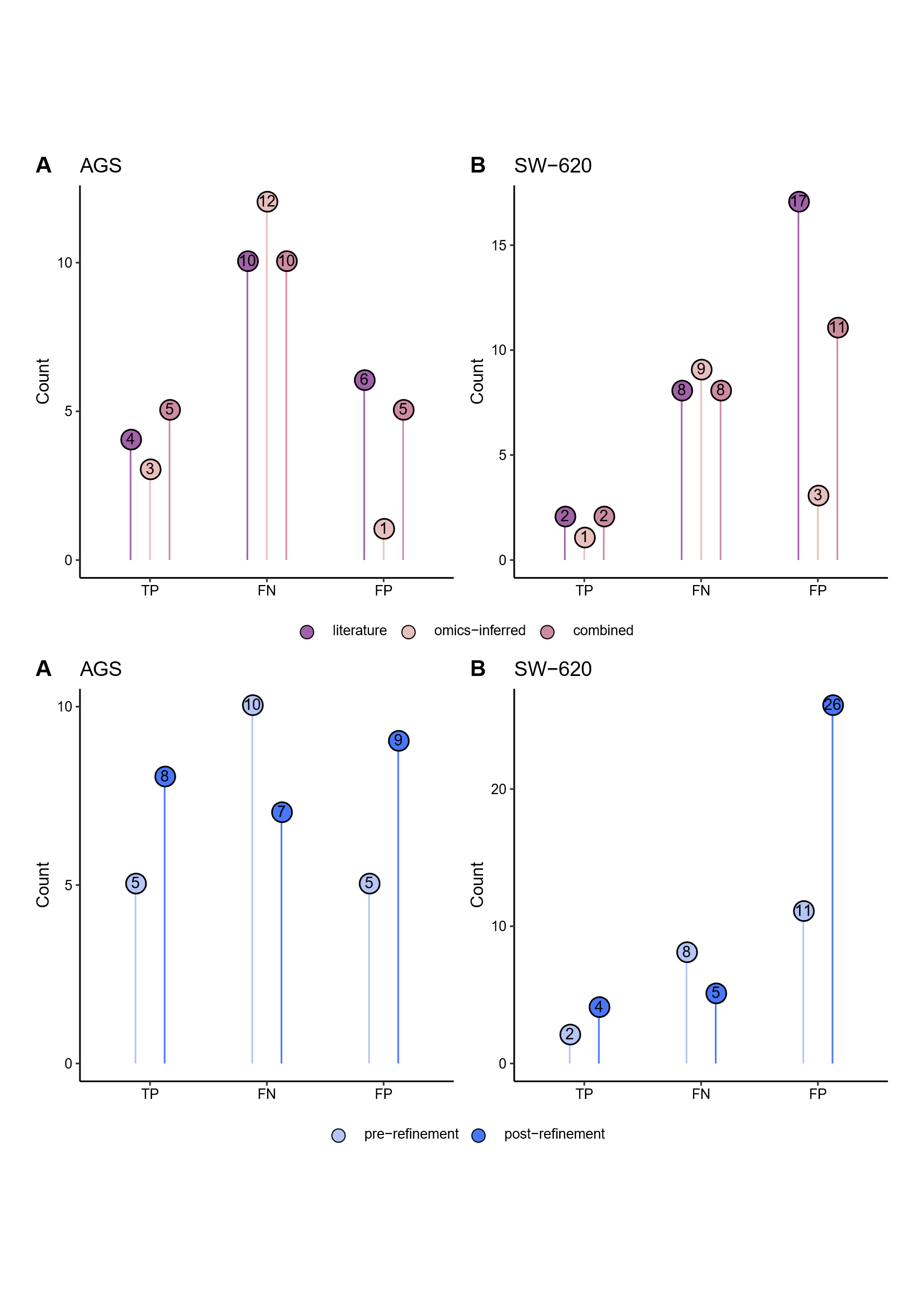


Supplementary Figure 2 - Comparison of predictions of PKN using different activity profiles.

(A) AGS and (B) SW-620. Numbers in dots indicate observations for true positives (TP), false negatives (FN) and false positives (FP).


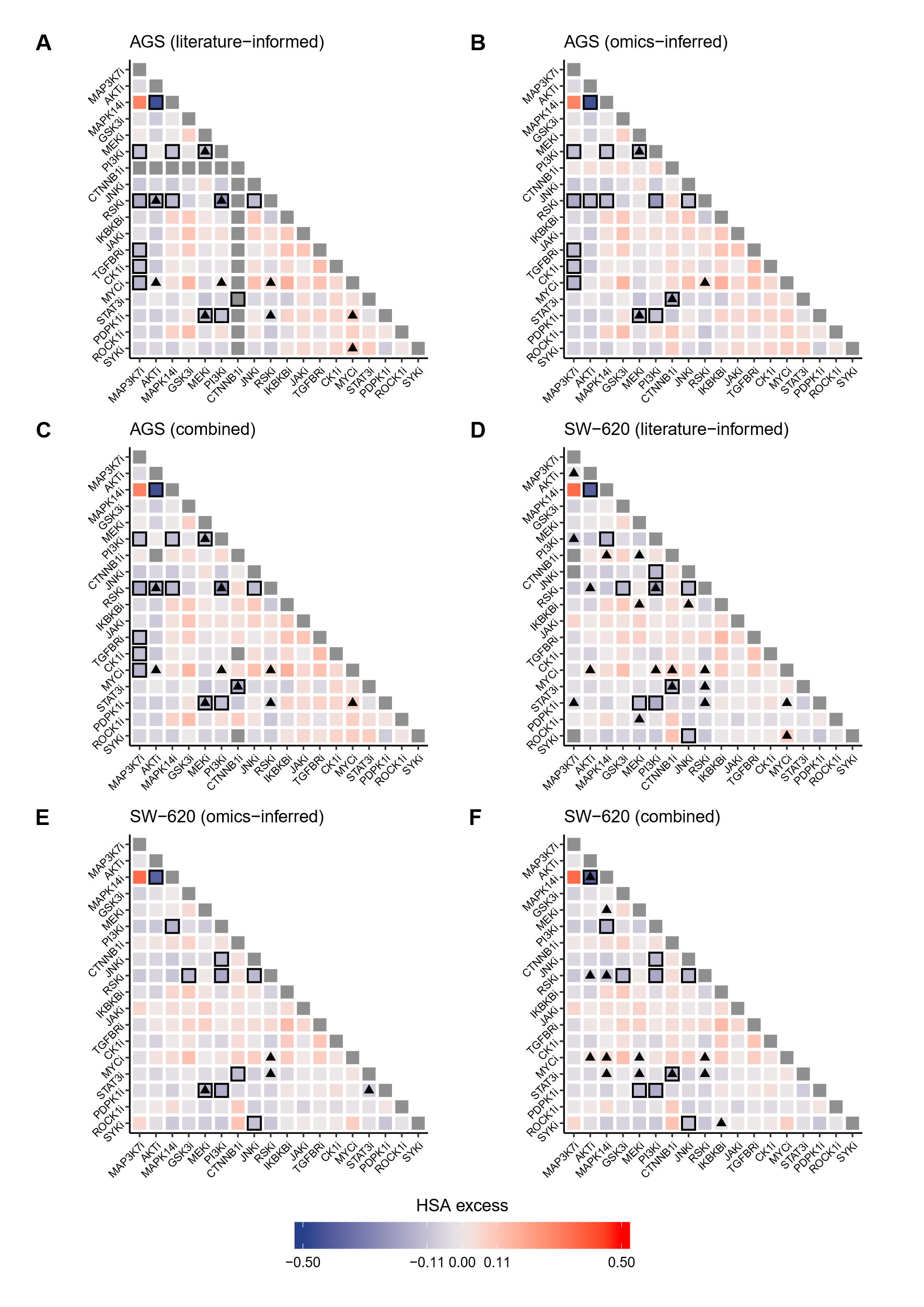


Supplementary Figure 3 – Predictions of AGS and SW-620 models using different activity profiles.

(A) literature-informed AGS model, (B) Paradigm-informed AGS model, (C) AGS model informed by combined baseline data, (D) literature-derived SW-620 model, (E) Paradigm-informed SW-620 model and (F) SW-620 model informed by combined baseline data. Predicted synergistic combinations are indicated by symbols in the plot. Combinations that could not be predicted are indicated in dark grey. Observed synergies in the testing data are indicated by a black frame. Tiles are colored according to Highest Single Agent (HSA) score with more synergistic combinations in blue and more antagonistic combinations colored in red.


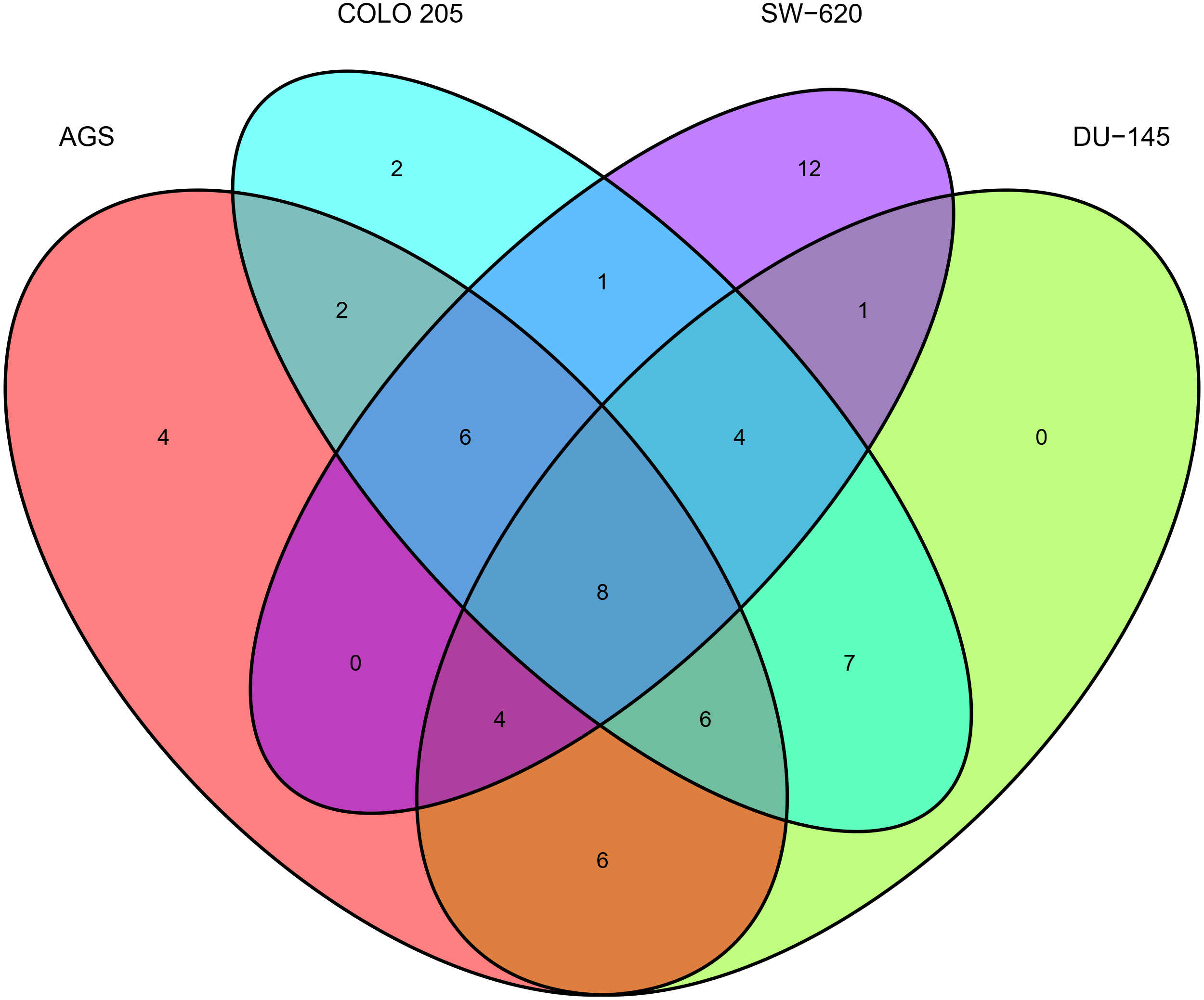


Supplementary Figure 4 - Venn diagram of top 36 high-influential nodes per cell lines.


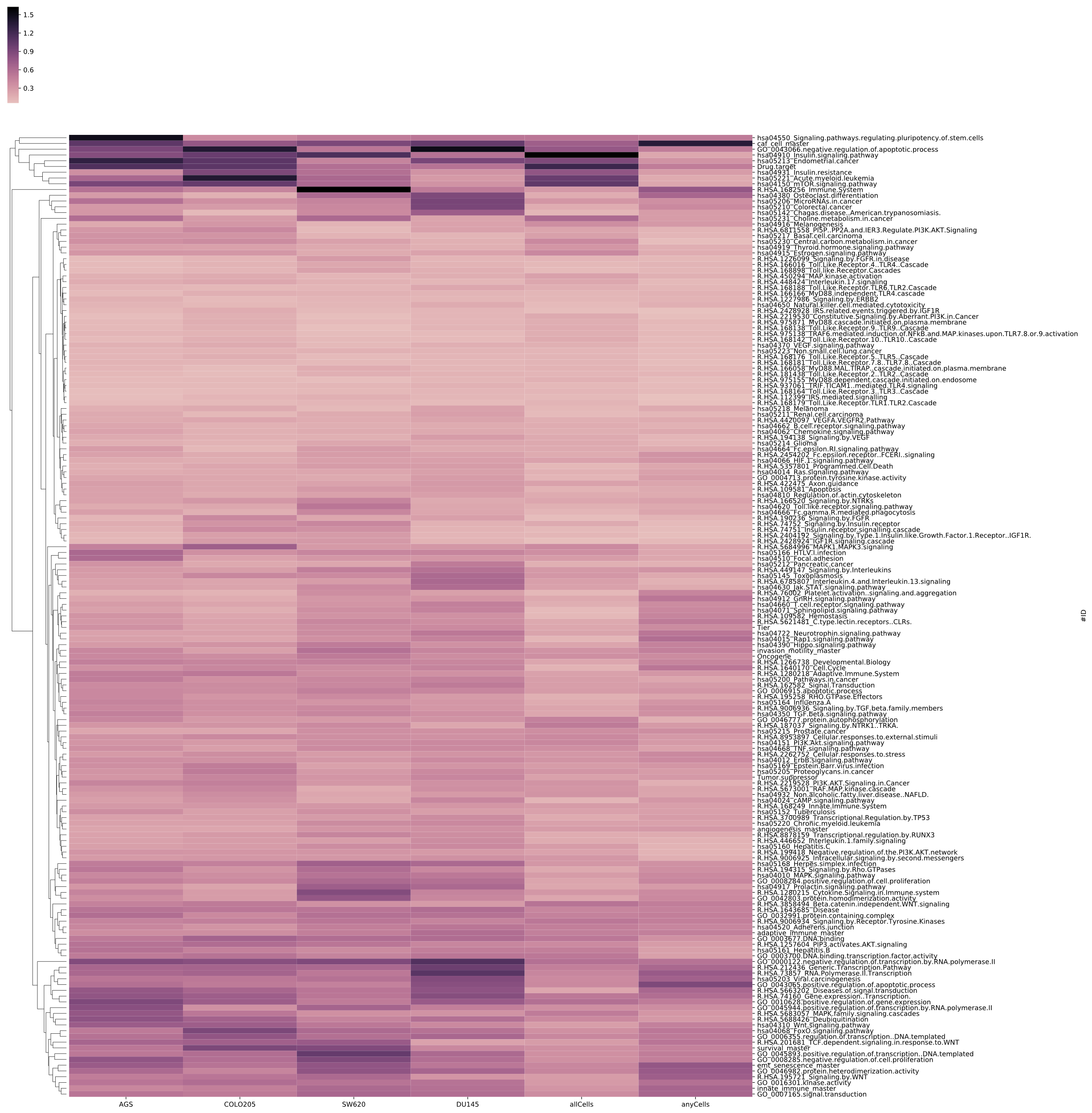


Supplementary Figure 5 - Biological feature importance.

Gini importance for biological features used by random forest to classify influential nodes in each cell lines, for all cell lines or of nodes influential in any cell lines.


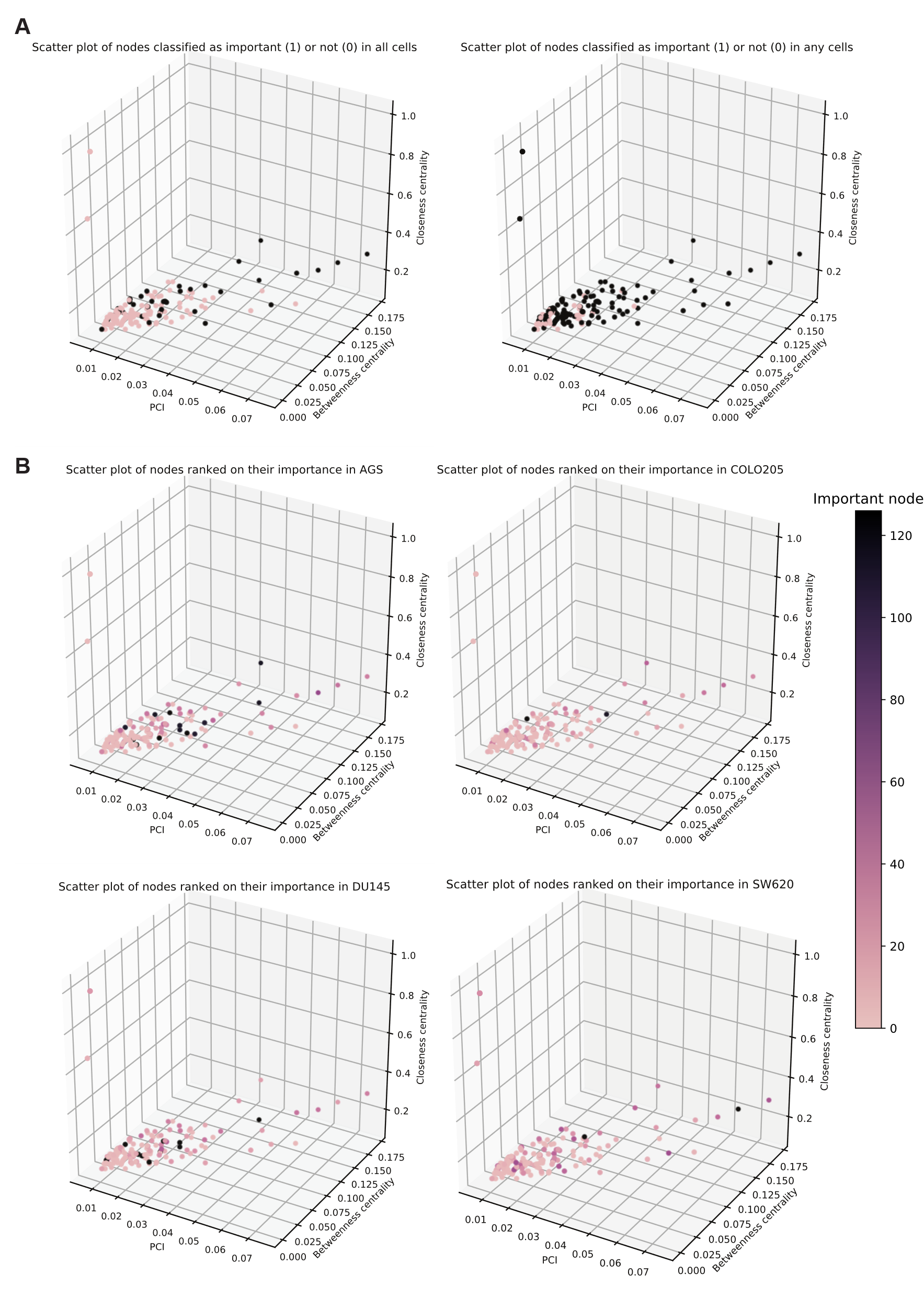


Supplementary Figure 6 - Scatter plot for PCI, Betweenness centrality and Closeness centrality distributions of high- and low-importance nodes.

(A) all or any cell line (color: dark - high-influence, light - low-influence) and (B) in AGS, COLO 205, DU-145 and COLO 205 cells (nodes ranked by importance).


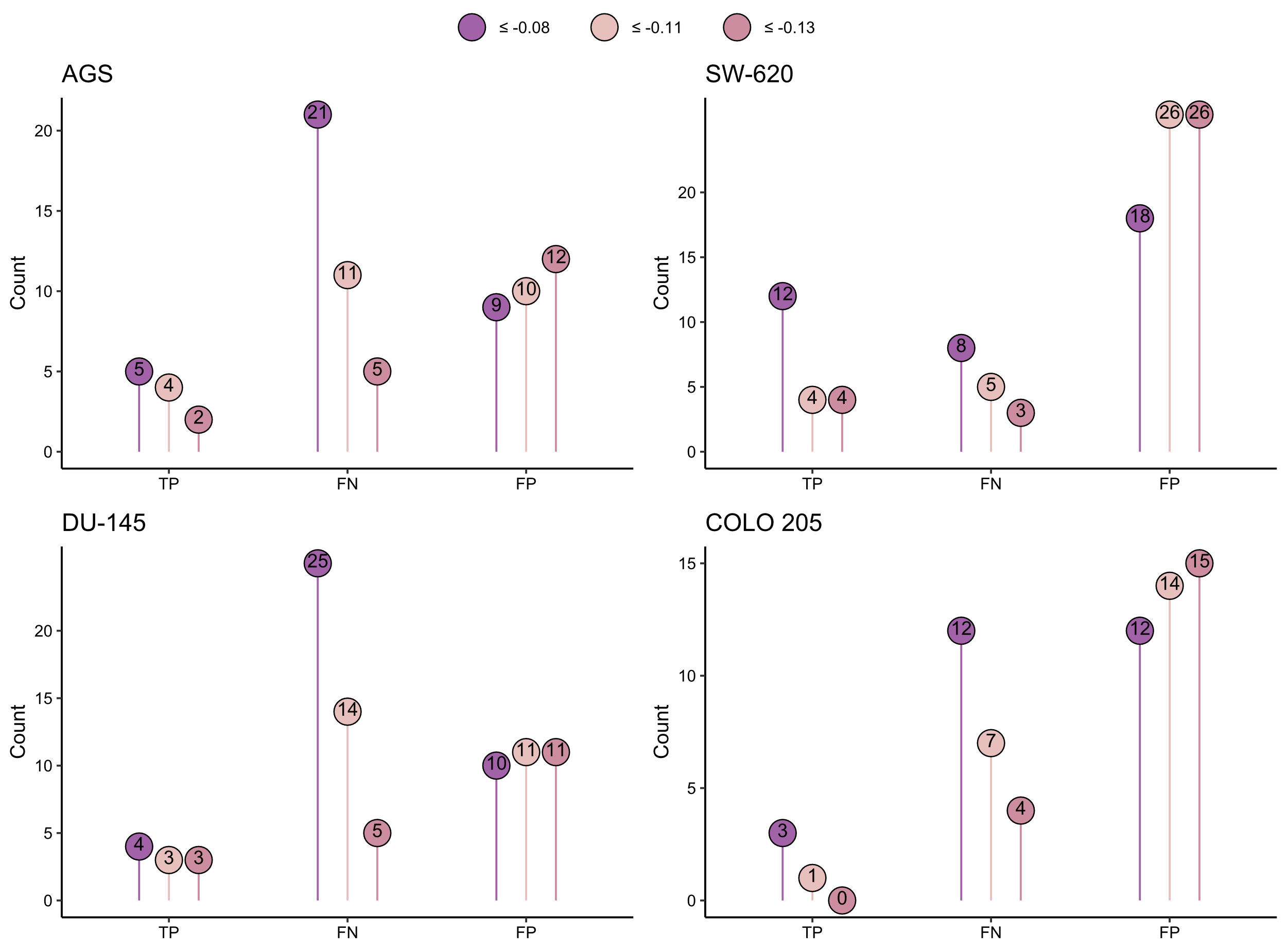


Supplementary Figure 7 – Model predictions for different synergy cut-offs.

Numbers in dots indicate observations for true positives (TP), false negatives (FN) and false positives (FP) when using different HSA cut-off for synergy calling.

Supplementary Tables

[Supplementary Table 1 – Logical rules for phenotypic output nodes. 15](#_Toc42120665)

[Supplementary Table 2 - Exceptions from generic logical rules. 15](#_Toc42120666)

[Supplementary Table 3 - List of nodes per cell line for which the omics-inferred and literature derived state contradicted each other. 16](#_Toc42120667)

[Supplementary Table 4 - Modified logical rules for literature informed AGS cell line model. 17](#_Toc42120668)

[Supplementary Table 5 - Modified logical rules for literature informed SW-620 cell line model. 18](#_Toc42120669)

[Supplementary Table 6 - Modified logical rules for omics-informed AGS cell line model. 19](#_Toc42120670)

[Supplementary Table 7 - Modified logical rules for omics-informed SW-620 cell line model. 21](#_Toc42120671)

[Supplementary Table 8 - Modified logical rules for combined AGS cell line model. 22](#_Toc42120672)

[Supplementary Table 9 - Modified logical rules for combined SW-620 cell line model. 24](#_Toc42120673)

[Supplementary Table 10 - Modified logical rules for combined DU-145 cell line model (refined network). 25](#_Toc42120674)

[Supplementary Table 11 - Modified logical rules for combined COLO 205 model (refined network). 26](#_Toc42120675)

[Supplementary Table 12 – Synergy predictions for the AGS cell line using PKN informed with different activity profiles. 28](#_Toc42120676)

[Supplementary Table 13 - Synergy predictions for the SW-620 cell line using PKN informed with different activity profiles. 28](#_Toc42120677)

[Supplementary Table 14 - Synergy predictions for the AGS cell line using subset of combined baseline data. 29](#_Toc42120678)

[Supplementary Table 15 – Synergy predictions for the SW-620 cell line using subset of combined baseline data. 29](#_Toc42120679)

[Supplementary Table 16 - Synergy predictions for the DU-145 cell line using subset of combined baseline data. 30](#_Toc42120680)

[Supplementary Table 17 - Synergy predictions for the COLO 205 cell line using subset of combined baseline data. 31](#_Toc42120681)

[Supplementary Table 18 - List of top 36 high-influence nodes per cell line ordered by number of affected combinations (high to low). 31](#_Toc42120682)

[Supplementary Table 19 - Modified logical rules for high-influence AGS cell model. 33](#_Toc42120683)

[Supplementary Table 20 - Modified rules in low-influence AGS cell model. 33](#_Toc42120684)

[Supplementary Table 21 - Modified rules for high-influence DU-145 model. 34](#_Toc42120685)

[Supplementary Table 22 - Modified rules for low-influence DU-145 model. 35](#_Toc42120686)

[Supplementary Table 23 - Modified rules for high-influence SW-620 model. 36](#_Toc42120687)

[Supplementary Table 24 - Modified rules for low-influence SW-620 model. 37](#_Toc42120688)

[Supplementary Table 25 - Modified logical rules for high-influence COLO 205 model. 37](#_Toc42120689)

[Supplementary Table 26 - Modified logical rules for low-influence COLO 205 model. 38](#_Toc42120690)

[Supplementary Table 27 - Model predictions using different HSA synergy cut-offs for each cell line. 39](#_Toc42120691)

Supplementary Table 1 – Logical rules for phenotypic output nodes.

The AND operator is represented by &, OR by |, and NOT by !.

| Node | Value | Rule |
| --- | --- | --- |
| Prosurvival | 1 | (MYC & !CCND1 & !RSK_f) \| (CCND1 & !MYC & !RSK_f) \| (RSK_f & !MYC & !CCND1) |
|  | 2 | (MYC & CCND1 & !RSK_f) \| (MYC & RSK_f & !CCND1) \| (RSK_f & CCND1 & !MYC) |
|  | 3 | MYC & CCND1 & RSK_f |
| Antisurvival | 1 | (FOXO_f & !CASP3 & !ISGF3_c) \| (CASP3 & !FOXO_f & !ISGF3_c) \| (ISGF3_c & !CASP3 & !FOXO_f) |
|  | 2 | (FOXO_f & CASP3 & !ISGF3_c) \| (FOXO_f & ISGF3_c & !CASP3) \| (ISGF3_c & CASP3 & !FOXO_f) |
|  | 3 | FOXO_f & CASP3 & ISGF3_c |

Supplementary Table 2 - Exceptions from generic logical rules.

| Node | Rule | Reference |
| --- | --- | --- |
| CBPp300_c | CREBBP & EP300 & !TP53 | PMID: 11559745, 10207072 |
| BTRC | CK1_f & AXIN1 & GSK3_f & !LRP_f | PMID: 23343194 |
| ISGF3_c | STAT1 & STAT2 | PMID: 8943351 |
| TGFB1 | (JUN & FOS) \| (NFkB_f & JUN) | PMID: 10843986; 2108318 |
| BAD | !RSK_f & !AKT_f | PMID: 16226704 |
| ATF2 | (MAPK14 & JNK_f) \| (ERK_f & MAPK14) | PMID: 12110590 |
| AP1_c | (FOS & JUN) \| (ATF2 & JUN) \| (SMAD3 & SMAD4 & ATF2) \| (SMAD3 & FOS) \| (SMAD3 & JUN) \| (SMAD3 & SMAD4) | PMID:10085140; 9732876; 20029425 |
| NFKB_f | (IKBKB \| CHUK \| MSK_f) & REL_f | PMID: 16382138 |
| SKP2 | (EP300 \| CCND1) & ERK_f | PMID: 16286470 |
| SMAD6_g | (SMAD2 \| SMAD3) & SMAD4 | PMID: 22836090, 21643690 |
| SMAD7_g | (SMAD2 \| SMAD3) & SMAD4 | PMID: 22836090, 21643690 |
| CTNNB1 | CHUK \| !BTRC | PMID: 16616828 |
| AKT_f | ((mTORC2_c \| ILK) & PDPK1) & !PPP1CA | PMID: 15209375 |

Supplementary Table 3 - List of nodes per cell line for which the omics-inferred and literature derived state contradicted each other.

The activity for these proteins in the combined activity profile and their activity in the tailored model are listed. PKN = Prior Knowledge Network.

| Cell line | Nodes | PKN | Omics-inferred | Literature | Combined | Activity in Model |
| --- | --- | --- | --- | --- | --- | --- |
| AGS | BCL2 | 0 | 0 | 1 | 1 | 1 |
|  | CASP8 | 1 | 1 | 0 | 0 | 0 |
|  | GSK3_f | 0 | 1 | 0 | 0 | 0 |
|  | JNK_f | 0 | 1 | 0 | 0 | 0 |
|  | KRAS | 0 | 0 | 1 | 1 | 1 |
|  | PIK3CA | 1 | 0 | 1 | 1 | 1 |
|  | RHOA | 0 | 0 | 1 | 1 | 1 |
|  | S6K_f | 0 | 0 | 1 | 1 | 1 |
|  | SOS1 | 0 | 0 | 1 | 1 | 1 |
| SW-620 | CASP3 | 1 | 1 | 0 | 0 | 0 |
|  | CASP8 | 1 | 1 | 0 | 0 | 0 |
|  | CASP9 | 0 | 1 | 0 | 0 | 0 |
|  | MAP3K7 | 0 | 0 | 1 | 1 | 1 |
|  | MAPK14 | 1 | 0 | 1 | 1 | 1 |
|  | PIK3CA | 1 | 0 | 1 | 1 | 1 |
|  | SMAD4 | 0 | 1 | 0 | 0 | 0 |
| COLO 205 | CASP8 | 1 | 1 | 0 | 0 | 0 |
|  | CYCS | 0 | 1 | 0 | 0 | 0 |
|  | ERK_f | 0 | 0 | 1 | 1 | 1 |
|  | GSK3_f | 0 | 1 | 0 | 0 | 0 |
| DU-145 | ATF2 | 0 | 0 | 1 | 1 | 1 |
|  | FOXO_f | 2 | 1 | 0 | 0 | 1 |
|  | GSK3_f | 0 | 1 | 0 | 0 | 0 |
|  | MAPK14 | 1 | 0 | 1 | 1 | 1 |
|  | MDM2 | 1 | 0 | 1 | 1 | 1 |
|  | NFKB_f | 1 | - | 1 | 1 | 1 |
|  | PIK3CA | 1 | 0 | 1 | 1 | 1 |
|  | RHOA | 0 | 0 | 1 | 1 | 1 |
|  | S6K_f | 0 | 0 | 1 | 1 | 1 |
|  | SMAD2 | 0 | 1 | 0 | 0 | 1 |
|  | SMAD3 | 0 | 1 | 0 | 0 | 0 |

Supplementary Table 4 - Modified logical rules for literature informed AGS cell line model.

| Node | Generic rule | Rule in literature informed AGS model |
| --- | --- | --- |
| AXIN^1^ | GSK3_f & !(LRP_f \| PPP1CA \| PPM1A) | GSK3_f \| !(LRP_f \| PPP1CA \| PPM1A) |
| DKK_g^1^ | TCF7_f & !MYC | TCF7_f \| !MYC |
| DUSP1 | (MAPK14 \| DUSP1_g \| MSK_f) & !SKP2 | MAPK14 \| DUSP1_g \| MSK_f \| !SKP2 |
| ERK_f | MEK_f & !(PPP1CA \| DUSP6) | MEK_f \| !(PPP1CA \| DUSP6) |
| GAB_f | GRB2 & !ERK_f | GRB2 \| !ERK_f |
| IRAK^2^ | ILR_f & !SOCS1 | ILR_f \| !SOCS1 |
| MAPK14 | MAP2K3 \| MAP2K4 \| !DUSP1 | (MAP2K3 \| MAP2K4) & !DUSP1 |
| MDM2 | (AKT_f \| PPP1CA \| MDM2_g \| MAPKAPK2) & !S6K_f | (AKT_f \| PPP1CA \| MDM2_g \| MAPKAPK2) \| !S6K_f |
| MEK_f | (RAF_f \| MAP3K8) & !ERK_f | (RAF_f \| MAP3K8) \| !ERK_f |
| RAC_f | (DVL_f \| mTORC2_c \| VAV1 \| TIAM1) & !ARHGAP24 | (DVL_f \| mTORC2_c \| VAV1 \| TIAM1) \| !ARHGAP24 |
| RAF_f^1^ | KRAS & !(AKT_f \| RHEB \| ERK_f) | KRAS \| !(AKT_f \| RHEB \| ERK_f) |
| REL_f^2^ | (CBPp300_c \| IKBKB \| MSK_f) & !STAT1 | CBPp300_c \| IKBKB \| MSK_f \| !STAT1 |
| RHOA | DAAM1 & !(RAC_f \| RND3 \| SMURF1 \| PARD6A) | DAAM1 \| !(RAC_f \| RND3 \| SMURF1 \| PARD6A) |
| RTPK_f | (MMP_f \| RTPK_g) & !(MAPK14 \| MEK_f) | MMP_f \| RTPK_g \| !(MAPK14 \| MEK_f) |
| SMAD4^1^ | (SMAD1 \| ERK_f \| SMAD5 \| SMAD2 \| PIAS1 \| SMAD3) & !(SMAD6 \| SKI \| SMURF1 \| SMAD7) | SMAD1 \| ERK_f \| SMAD5 \| SMAD2 \| PIAS1 \| SMAD3 \| !(SMAD6 \| SKI \| SMURF1 \| SMAD7) |
| SOS1 | (GRB2 \| PLCG1) & !ERK_f | GRB2 \| PLCG1 \| !ERK_f |
| SRC | RTPK_f & !CSK | RTPK_f \| !CSK |
| TCF7_f^1^ | CTNNB1 & !NLK | CTNNB1 \| !NLK |
| ^1^changed that a stable state can be reached  ^2^ STAT1 cannot be turned off since several activators of that node are supposed to be active and the inhibitor inactive. MAPK14 has more papers describing the node to be inactive than STAT1. In order to inhibit STAT1s function, the downstream targets of that nodes were inactivated. | | |

Supplementary Table 5 - Modified logical rules for literature informed SW-620 cell line model.

| Node | Generic rule | Rule in literature informed SW-620 model |
| --- | --- | --- |
| CFLAR | AKT_f & !ITCH | AKT_f \| !ITCH |
| DVL_f | (FZD_f \| SMAD1) & !ITCH | FZD_f \| SMAD1 \| !ITCH |
| ERK_f | MEK_f & !(PPP1CA \| DUSP6) | MEK_f \| !(PPP1CA \| DUSP6) |
| GAB_f | GRB2 & !ERK_f | GRB2 \| !ERK_f |
| IKBKB | MAP3K7 & !(TP53 \| PLK1 \| PPM1A) | MAP3K7 \| !(TP53 \| PLK1 \| PPM1A) |
| MAPK14 | MAP2K3 \| MAP2K4 \| !DUSP1 | (MAP2K3 \| MAP2K4) & !DUSP1 |
| MEK_f | (RAF_f \| MAP3K8) & !ERK_f | RAF_f \| MAP3K8 \| !ERK_f |
| RAC_f | (DVL_f \| mTORC2_c \| VAV1 \| TIAM1) & !ARHGAP24 | DVL_f \| mTORC2_c \| VAV1 \| TIAM1 \| !ARHGAP24 |
| RHOA | DAAM1 & !(RAC_f \| RND3 \| SMURF1 \| PARD6A) | DAAM1 \| !(RAC_f \| RND3 \| SMURF1 \| PARD6A) |
| RTPK_f | (MMP_f \| RTPK_g) & !(MAPK14 \| MEK_f) | MMP_f \| RTPK_g \| !(MAPK14 \| MEK_f) |
| SMAD3 | (TGFBR1 \| JNK_f \| MAPK14 \| ACVR1) & !(SMAD6 \| GSK3_f \| AKT_f \| ERK_f \| PPM1A \| SKI \| SMAD7) | TGFBR1 \| JNK_f \| MAPK14 \| ACVR1 \| !(SMAD6 \| GSK3_f \| AKT_f \| ERK_f \| PPM1A \| SKI \| SMAD7) |
| SMAD7^1^ | (EP300 \| SMAD7_g \| SMURF1) & !(AXIN1 \| SMURF2 \| ITCH) | EP300 \| SMAD7_g \| SMURF1 \| !(AXIN1 \| SMURF2 \| ITCH) |
| SRC | RTPK_f & !CSK | RTPK_f \| !CSK |
| TAB_f | TRAF6 & !MAPK14 | TRAF6 \| !MAPK14 |
| ^1^changed that a stable state can be reached | | |

Supplementary Table 6 - Modified logical rules for omics-informed AGS cell line model.

| Node | Generic rule | Rule in omics-informed AGS model |
| --- | --- | --- |
| AXIN1 | GSK3_f & !(LRP_f \| PPP1CA \| PPM1A) | GSK3_f \| !(LRP_f \| PPP1CA \| PPM1A) |
| BTRC | CK1_f & AXIN1 & GSK3_f & !LRP_f | (CK1_f & AXIN1 & GSK3_f) \| !LRP_f |
| CDC42 | SRC & !ARHGAP24 | SRC \| !ARHGAP24 |
| DKK_g | TCF7_f & !MYC | TCF7_f \| !MYC |
| DUSP1 | (MAPK14 \| DUSP1_g \| MSK_f) & !SKP2 | MAPK14 \| DUSP1_g \| MSK_f \| !SKP2 |
| DVL_f | (FZD_f \| SMAD1) & !ITCH | FZD_f \| SMAD1 \| !ITCH |
| ERK_f | MEK_f & !(PPP1CA \| DUSP6) | MEK_f \| !(PPP1CA \| DUSP6) |
| GAB_f | GRB2 & !ERK_f | GRB2 \| !ERK_f |
| IKBKB | MAP3K7 & !(TP53 \| PLK1 \| PPM1A) | MAP3K7 \| !(TP53 \| PLK1 \| PPM1A) |
| IRAK1 | ILR_f & !SOCS1 | ILR_f \| !SOCS1 |
| JNK_f | (MAP2K4 \| MAP2K7 \| PAK1) & !DUSP1 | MAP2K4 \| MAP2K7 \| PAK1 \| !DUSP1 |
| LIMK2 | ROCK1 & !PRKCD | ROCK1 \| !PRKCD |
| MAPK14 | MAP2K3 \| MAP2K4 \| !DUSP1 | (MAP2K3 \| MAP2K4) & !DUSP1 |
| MDM2 | (AKT_f \| PPP1CA \| MDM2_g \| MAPKAPK2) & !S6K_f | AKT_f \| PPP1CA \| MDM2_g \| MAPKAPK2 \| !S6K_f |
| MEK_f | (RAF_f \| MAP3K8) & !ERK_f | RAF_f \| MAP3K8 \| !ERK_f |
| RAC_f | (DVL_f \| mTORC2_c \| VAV1 \| TIAM1) & !ARHGAP24 | DVL_f \| mTORC2_c \| VAV1 \| TIAM1 \| !ARHGAP24 |
| RAF_f | KRAS & !(AKT_f \| RHEB \| ERK_f) | KRAS \| !(AKT_f \| RHEB \| ERK_f) |
| REL_f | (CBPp300_c \| IKBKB \| MSK_f) & !STAT1 | CBPp300_c \| IKBKB \| MSK_f \| !STAT1 |
| RTPK_f | (MMP_f \| RTPK_g) & !(MAPK14 \| MEK_f) | MMP_f \| RTPK_g \| !(MAPK14 \| MEK_f) |
| SMAD3 | (TGFBR1 \| JNK_f \| MAPK14 \| ACVR1) & !(SMAD6 \| GSK3_f \| AKT_f \| ERK_f \| PPM1A \| SKI \| SMAD7) | TGFBR1 \| JNK_f \| MAPK14 \| ACVR1 \| !(SMAD6 \| GSK3_f \| AKT_f \| ERK_f \| PPM1A \| SKI \| SMAD7) |
| SRC | RTPK_f & !CSK | RTPK_f \| !CSK |
| TCF7_f | CTNNB1 & !NLK | CTNNB1 \| !NLK |
| TGFBR1 | TGFBR2 & !(SMAD6 \| SMURF1 \| SMURF2 \| SMAD7) | TGFBR2 \| !(SMAD6 \| SMURF1 \| SMURF2 \| SMAD7) |

Supplementary Table 7 - Modified logical rules for omics-informed SW-620 cell line model.

| Node | Generic rule | Rule in omics-informed SW-620 model |
| --- | --- | --- |
| AKT_f | (mTORC2_c \| ILK) & PDPK1 & !PPP1CA | ((mTORC2_c \| ILK) & PDPK1) \| !PPP1CA |
| AXIN1 | GSK3_f & !(LRP_f \| PPP1CA \| PPM1A) | GSK3_f \| !(LRP_f \| PPP1CA \| PPM1A) |
| CDC42 | SRC & !ARHGAP24 | SRC \| !ARHGAP24 |
| CYCS | BAX & !BCL2 | BAX \| !BCL2 |
| DKK_g | TCF7_f & !MYC | TCF7_f \| !MYC |
| DUSP1 | (MAPK14 \| DUSP1_g \| MSK_f) & !SKP2 | MAPK14 \| DUSP1_g \| MSK_f \| !SKP2 |
| DVL_f | (FZD_f \| SMAD1) & !ITCH | FZD_f \| SMAD1 \| !ITCH |
| EP300 | AKT_f & !(PRKCD \| SKI) | AKT_f \| !(PRKCD \| SKI) |
| ERK_f | MEK_f & !(PPP1CA \| DUSP6) | MEK_f \| !(PPP1CA \| DUSP6) |
| GAB_f | GRB2 & !ERK_f | GRB2 \| !ERK_f |
| IKBKB | MAP3K7 & !(TP53 \| PLK1 \| PPM1A) | MAP3K7 \| !(TP53 \| PLK1 \| PPM1A) |
| IRAK1 | ILR_f & !SOCS1 | ILR_f \| !SOCS1 |
| JNK_f | (MAP2K4 \| MAP2K7 \| PAK1) & !DUSP1 | MAP2K4 \| MAP2K7 \| PAK1 \| !DUSP1 |
| MAPK14 | MAP2K3 \| MAP2K4 \| !DUSP1 | (MAP2K3 \| MAP2K4) & !DUSP1 |
| MDM2 | (AKT_f \| PPP1CA \| MDM2_g \| MAPKAPK2) & !S6K_f | AKT_f \| PPP1CA \| MDM2_g \| MAPKAPK2 \| !S6K_f |
| MEK_f | (RAF_f \| MAP3K8) & !ERK_f | RAF_f \| MAP3K8 \| !ERK_f |
| PDPK1 | PIK3CA & !PTEN | PIK3CA \| !PTEN |
| PPP1CA | SMAD7 & !RTPK_f | SMAD7 \| !RTPK_f |
| PTEN | (PTEN_g \| ROCK1) & !(SRC \| CBPp300_c \| GSK3_f) | PTEN_g \| ROCK1 \| !(SRC \| CBPp300_c \| GSK3_f) |
| PTEN | PTEN_g \| ROCK1 \| !(SRC \| CBPp300_c \| GSK3_f) | (PTEN_g \| ROCK1) & !(SRC \| CBPp300_c \| GSK3_f) |
| RAF_f | KRAS & !(AKT_f \| RHEB \| ERK_f) | KRAS \| !(AKT_f \| RHEB \| ERK_f) |
| REL_f | CBPp300_c \| IKBKB \| MSK_f) & !STAT1 | CBPp300_c \| IKBKB \| MSK_f \| !STAT1 |
| RTPK_f | (MMP_f \| RTPK_g) & !(MAPK14 \| MEK_f) | MMP_f \| RTPK_g \| !(MAPK14 \| MEK_f) |
| SMAD1 | ACVR1 & !(GSK3_f \| ERK_f \| PPM1A \| SKI \| SMURF1 \| SMAD6) | ACVR1 \| !(GSK3_f \| ERK_f \| PPM1A \| SKI \| SMURF1 \| SMAD6) |
| SMAD3 | (TGFBR1 \| JNK_f \| MAPK14 \| ACVR1) & !(SMAD6 \| GSK3_f \| AKT_f \| ERK_f \| PPM1A \| SKI \| SMAD7) | TGFBR1 \| JNK_f \| MAPK14 \| ACVR1 \| !(SMAD6 \| GSK3_f \| AKT_f \| ERK_f \| PPM1A \| SKI \| SMAD7) |
| SMAD4 | (SMAD1 \| ERK_f \| SMAD5 \| SMAD2 \| PIAS1 \| SMAD3) & !(SMAD6 \| SKI \| SMURF1 \| SMAD7) | SMAD1 \| ERK_f \| SMAD5 \| SMAD2 \| PIAS1 \| SMAD3 \| !(SMAD6 \| SKI \| SMURF1 \| SMAD7) |
| SMAD7 | (EP300 \| SMAD7_g \| SMURF1) & !(AXIN1 \| SMURF2 \| ITCH) | EP300 \| SMAD7_g \| SMURF1 \| !(AXIN1 \| SMURF2 \| ITCH) |
| SOS1^1^ | (GRB2 \| PLCG1) & !ERK_f | GRB2 \| PLCG1 \| !ERK_f |
| SRC | RTPK_f & !CSK | RTPK_f \| !CSK |
| STAT3 | (SRC \| IRAK1 \| JAK_f \| mTORC1_c \| MAPK14 \| JNK_f \| ERK_f \| PRKCD) & !PPP1CA | SRC \| IRAK1 \| JAK_f \| mTORC1_c \| MAPK14 \| JNK_f \| ERK_f \| PRKCD \| !PPP1CA |
| TGFBR1 | TGFBR2 & !(SMAD6 \| SMURF1 \| SMURF2 \| SMAD7) | TGFBR2 \| !(SMAD6 \| SMURF1 \| SMURF2 \| SMAD7) |
| TGFBR2 | TGFB1 & !(SMURF1 \| SMURF2) | TGFB1 \| !(SMURF1 \| SMURF2) |
| TSC_f | GSK3_f & !(AKT_f \| ERK_f \| IKBKB \| RSK_f) | GSK3_f \| !(AKT_f \| ERK_f \| IKBKB \| RSK_f) |
| ^1^changed that a stable state can be reached | | |

Supplementary Table 8 - Modified logical rules for combined AGS cell line model.

| Node | Generic rule | Rule in combined AGS model |
| --- | --- | --- |
| AXIN1 | GSK3_f & !(LRP_f \| PPP1CA \| PPM1A) | GSK3_f \| !(LRP_f \| PPP1CA \| PPM1A) |
| BTRC | CK1_f & AXIN1 & GSK3_f & !LRP_f | (CK1_f & AXIN1 & GSK3_f) \| !LRP_f |
| CDC42 | SRC & !ARHGAP24 | SRC \| !ARHGAP24 |
| DKK_g | TCF7_f & !MYC | TCF7_f \| !MYC |
| ERK_f | MEK_f & !(PPP1CA \| DUSP6) | MEK_f \| !(PPP1CA \| DUSP6) |
| GAB_f | GRB2 & !ERK_f | GRB2 \| !ERK_f |
| IKBKB | MAP3K7 & !(TP53 \| PLK1 \| PPM1A) | MAP3K7 \| !(TP53 \| PLK1 \| PPM1A) |
| IRAK1 | ILR_f & !SOCS1 | ILR_f \| !SOCS1 |
| MAPK14 | MAP2K3 \| MAP2K4 \| !DUSP1 | (MAP2K3 \| MAP2K4) & !DUSP1 |
| MDM2 | (AKT_f \| PPP1CA \| MDM2_g \| MAPKAPK2) & !S6K_f | AKT_f \| PPP1CA \| MDM2_g \| MAPKAPK2 \| !S6K_f |
| MEK_f | (RAF_f \| MAP3K8) & !ERK_f | RAF_f \| MAP3K8 \| !ERK_f |
| RAC_f | (DVL_f \| mTORC2_c \| VAV1 \| TIAM1) & !ARHGAP24 | DVL_f \| mTORC2_c \| VAV1 \| TIAM1 \| !ARHGAP24 |
| RAF_f | KRAS & !(AKT_f \| RHEB \| ERK_f) | KRAS \| !(AKT_f \| RHEB \| ERK_f) |
| REL_f | (CBPp300_c \| IKBKB \| MSK_f) & !STAT1 | CBPp300_c \| IKBKB \| MSK_f \| !STAT1 |
| RHOA | DAAM1 & !(RAC_f \| RND3 \| SMURF1 \| PARD6A) | DAAM1 \| !(RAC_f \| RND3 \| SMURF1 \| PARD6A) |
| RTPK_f | (MMP_f \| RTPK_g) & !(MAPK14 \| MEK_f) | MMP_f \| RTPK_g \| !(MAPK14 \| MEK_f) |
| SMAD3 | (TGFBR1 \| JNK_f \| MAPK14 \| ACVR1) & !(SMAD6 \| GSK3_f \| AKT_f \| ERK_f \| PPM1A \| SKI \| SMAD7) | TGFBR1 \| JNK_f \| MAPK14 \| ACVR1 \| !(SMAD6 \| GSK3_f \| AKT_f \| ERK_f \| PPM1A \| SKI \| SMAD7) |
| SMAD4 | (SMAD1 \| ERK_f \| SMAD5 \| SMAD2 \| PIAS1 \| SMAD3) & !(SMAD6 \| SKI \| SMURF1 \| SMAD7) | SMAD1 \| ERK_f \| SMAD5 \| SMAD2 \| PIAS1 \| SMAD3 \| !(SMAD6 \| SKI \| SMURF1 \| SMAD7) |
| SOS1 | (GRB2 \| PLCG1) & !ERK_f | GRB2 \| PLCG1 \| !ERK_f |
| SRC | RTPK_f & !CSK | RTPK_f \| !CSK |
| TCF7_f^1^ | CTNNB1 & !NLK | CTNNB1 \| !NLK |
| TGFBR1 | TGFBR2 & !(SMAD6 \| SMURF1 \| SMURF2 \| SMAD7) | TGFBR2 \| !(SMAD6 \| SMURF1 \| SMURF2 \| SMAD7) |
| TSC_f | GSK3_f & !(AKT_f \| ERK_f \| IKBKB \| RSK_f) | GSK3_f \| !(AKT_f \| ERK_f \| IKBKB \| RSK_f) |
| ^1^changed that a stable state can be reached | | |

Supplementary Table 9 - Modified logical rules for combined SW-620 cell line model.

| Node | Generic rule | Rule in combined SW-620 model |
| --- | --- | --- |
| AKT_f | (mTORC2_c \| ILK) & PDPK1 & !PPP1CA | ((mTORC2_c \| ILK) & PDPK1) \| !PPP1CA |
| AXIN1 | GSK3_f & !(LRP_f \| PPP1CA \| PPM1A) | GSK3_f \| !(LRP_f \| PPP1CA \| PPM1A) |
| CFLAR | AKT_f & !ITCH | AKT_f \| !ITCH |
| DKK_g | TCF7_f & !MYC | TCF7_f \| !MYC |
| DVL_f | (FZD_f \| SMAD1) & !ITCH | FZD_f \| SMAD1 \| !ITCH |
| EP300 | AKT_f & !(PRKCD \| SKI) | AKT_f \| !(PRKCD \| SKI) |
| ERK_f | MEK_f & !(PPP1CA \| DUSP6) | MEK_f \| !(PPP1CA \| DUSP6) |
| GAB_f | GRB2 & !ERK_f | GRB2 \| !ERK_f |
| IKBKB | MAP3K7 & !(TP53 \| PLK1 \| PPM1A)\| | MAP3K7 \| !(TP53 \| PLK1 \| PPM1A) |
| JAK_f | ILR_f & !(SOCS1 \| PTPN6) | ILR_f \| !(SOCS1 \| PTPN6) |
| LIMK2 | ROCK1 & !PRKCD | ROCK1 \| !PRKCD |
| MDM2 | (AKT_f \| PPP1CA \| MDM2_g \| MAPKAPK2) & !S6K_f | AKT_f \| PPP1CA \| MDM2_g \| MAPKAPK2 \| !S6K_f |
| MEK_f | (RAF_f \| MAP3K8) & !ERK_f | RAF_f \| MAP3K8 \| !ERK_f |
| PTEN | PTEN = (PTEN_g \| ROCK1) & !(SRC \| CBPp300_c \| GSK3_f) | PTEN_g \| ROCK1 \| !(SRC \| CBPp300_c \| GSK3_f) |
| RAF_f | KRAS & !(AKT_f \| RHEB \| ERK_f) | KRAS \| !(AKT_f \| RHEB \| ERK_f) |
| RTPK_f | (MMP_f \| RTPK_g) & !(MAPK14 \| MEK_f) | MMP_f \| RTPK_g \| !(MAPK14 \| MEK_f) |
| SMAD1 | ACVR1 & !(GSK3_f \| ERK_f \| PPM1A \| SKI \| SMURF1 \| SMAD6) | ACVR1 \| !(GSK3_f \| ERK_f \| PPM1A \| SKI \| SMURF1 \| SMAD6) |
| SMAD3 | (TGFBR1 \| JNK_f \| MAPK14 \| ACVR1) & !(SMAD6 \| GSK3_f \| AKT_f \| ERK_f \| PPM1A \| SKI \| SMAD7) | TGFBR1 \| JNK_f \| MAPK14 \| ACVR1 \| !(SMAD6 \| GSK3_f \| AKT_f \| ERK_f \| PPM1A \| SKI \| SMAD7) |
| SMAD7 | (EP300 \| SMAD7_g \| SMURF1) & !(AXIN1 \| SMURF2 \| ITCH) | EP300 \| SMAD7_g \| SMURF1 \| !(AXIN1 \| SMURF2 \| ITCH) |
| SOS1 | (GRB2 \| PLCG1) & !ERK_f | GRB2 \| PLCG1 \| !ERK_f |
| SRC | RTPK_f & !CSK | RTPK_f \| !CSK |
| STAT3 | (SRC \| IRAK1 \| JAK_f \| mTORC1_c \| MAPK14 \| JNK_f \| ERK_f \| PRKCD) & !PPP1CA | SRC \| IRAK1 \| JAK_f \| mTORC1_c \| MAPK14 \| JNK_f \| ERK_f \| PRKCD \| !PPP1CA |
| TAB_f | TRAF6 & !MAPK14 | TRAF6 \| !MAPK14 |
| TGFBR1 | TGFBR2 & !(SMAD6 \| SMURF1 \| SMURF2 \| SMAD7) | TGFBR2 \| !(SMAD6 \| SMURF1 \| SMURF2 \| SMAD7) |
| TGFBR2 | TGFB1 & !(SMURF1 \| SMURF2) | TGFB1 \| !(SMURF1 \| SMURF2) |
| TSC_f | GSK3_f & !(AKT_f \| ERK_f \| IKBKB \| RSK_f) | GSK3_f \| !(AKT_f \| ERK_f \| IKBKB \| RSK_f) |

Supplementary Table 10 - Modified logical rules for combined DU-145 cell line model (refined network).

| Node | Generic rule | Rule in combined DU-145 model |
| --- | --- | --- |
| AXIN1 | GSK3_f & !(LRP_f \| PPP1CA \| PPM1A) | GSK3_f \| !(LRP_f \| PPP1CA \| PPM1A) |
| BTRC | CK1_f & AXIN1 & GSK3_f & !LRP_f | (CK1_f & AXIN1 & GSK3_f) \| !LRP_f |
| CDC42 | SRC & !ARHGAP24 | SRC \| !ARHGAP24 |
| CFLAR | AKT_f & !ITCH | AKT_f \| !ITCH |
| CFLAR | AKT_f & !ITCH | AKT_f \| !ITCH |
| CTNNB1 | CHUK & !BTRC | CHUK \| !BTRC |
| DKK_g | TCF7_f & !MYC | TCF7_f \| !MYC |
| DVL_f | (FZD_f \| SMAD1) & !ITCH | FZD_f \| SMAD1 \| !ITCH |
| EP300 | AKT_f & !(PRKCD \| SKI) | AKT_f \| !(PRKCD \| SKI) |
| ERK_f | MEK_f & !(PPP1CA \| DUSP6) | MEK_f \| !(PPP1CA \| DUSP6) |
| GAB_f | GRB2 & !ERK_f | GRB2 \| !ERK_f |
| IKBKB | MAP3K7 & !(TP53 \| PLK1 \| PPM1A) | MAP3K7 \| !(TP53 \| PLK1 \| PPM1A) |
| JAK_f | ILR_f & !(SOCS1 \| PTPN6) | ILR_f \| !(SOCS1 \| PTPN6) |
| JNK_f | (MAP2K4 \| MAP2K7 \| PAK1) & !DUSP1 | MAP2K4 \| MAP2K7 \| PAK1 \| !DUSP1 |
| MDM2 | (AKT_f \| PPP1CA \| MDM2_g \| MAPKAPK2) & !S6K_f | AKT_f \| PPP1CA \| MDM2_g \| MAPKAPK2 \| !S6K_f |
| MEK_f | (RAF_f \| MAP3K8) & !ERK_f | RAF_f \| MAP3K8 \| !ERK_f |
| RAC_f | (DVL_f \| mTORC2_c \| VAV1 \| TIAM1) & !ARHGAP24 | DVL_f \| mTORC2_c \| VAV1 \| TIAM1 \| !ARHGAP24 |
| RAF_f | KRAS & !(AKT_f \| RHEB \| ERK_f) | KRAS \| !(AKT_f \| RHEB \| ERK_f) |
| RHOA | DAAM1 & !(RAC_f \| RND3 \| SMURF1 \| PARD6A) | DAAM1 \| !(RAC_f \| RND3 \| SMURF1 \| PARD6A) |
| RTPK_f | (MMP_f \| RTPK_g) & !(MAPK14 \| MEK_f) | MMP_f \| RTPK_g \| !(MAPK14 \| MEK_f) |
| SMAD4 | (SMAD1 \| ERK_f \| SMAD5 \| SMAD2 \| PIAS1 \| SMAD3) & !(SMAD6 \| SKI \| SMURF1 \| SMAD7) | SMAD1 \| ERK_f \| SMAD5 \| SMAD2 \| PIAS1 \| SMAD3 \| !(SMAD6 \| SKI \| SMURF1 \| SMAD7) |
| SRC | RTPK_f & !CSK | RTPK_f \| !CSK |
| TGFBR1 | TGFBR2 & !(SMAD6 \| SMURF1 \| SMURF2 \| SMAD7) | TGFBR2 \| !(SMAD6 \| SMURF1 \| SMURF2 \| SMAD7) |
| TSC_f | GSK3_f & !(AKT_f \| ERK_f \| IKBKB \| RSK_f) | GSK3_f \| !(AKT_f \| ERK_f \| IKBKB \| RSK_f) |

Supplementary Table 11 - Modified logical rules for combined COLO 205 model (refined network).

| Node | Generic rule | Rule in combined COLO 205 model |
| --- | --- | --- |
| AKT_f | (mTORC2_c \| ILK) & PIP3 & !PPP1CA | ((mTORC2_c \| ILK) & PIP3) \| !PPP1CA |
| AXIN1 | GSK3_f & !(LRP_f \| PPP1CA \| PPM1A) | GSK3_f \| !(LRP_f \| PPP1CA \| PPM1A) |
| BTRC | CK1_f & AXIN1 & GSK3_f & !LRP_f | (CK1_f & AXIN1 & GSK3_f) \| !LRP_f |
| CFLAR | AKT_f & !ITCH | AKT_f \| !ITCH |
| CTNNB1 | CHUK & !BTRC | CHUK \| !BTRC |
| DUSP1 | (MAPK14 \| DUSP1_g \| MSK_f) & !SKP2 | (MAPK14 \| DUSP1_g \| MSK_f) \| !SKP2 |
| EP300 | AKT_f & !(PRKCD \| SKI) | AKT_f \| !(PRKCD \| SKI) |
| ERK_f | MEK_f & !(PPP1CA \| DUSP6) | MEK_f \| !(PPP1CA \| DUSP6) |
| GAB_f | GRB2 & !ERK_f | GRB2 \| !ERK_f |
| IKBKB | MAP3K7 & !(TP53 \| PLK1 \| PPM1A) | MAP3K7 \| !(TP53 \| PLK1 \| PPM1A) |
| IRAK1 | ILR_f & !SOCS1 | ILR_f \| !SOCS1 |
| JNK_f | (MAP2K4 \| MAP2K7 \| PAK1) & !DUSP1 | MAP2K4 \| MAP2K7 \| PAK1 \| !DUSP1 |
| LIMK2 | ROCK1 & !PRKCD | ROCK1 \| !PRKCD |
| MDM2^1^ | (AKT_f \| PPP1CA \| MDM2_g \| MAPKAPK2) & !S6K_f | AKT_f \| PPP1CA \| MDM2_g \| MAPKAPK2 \| !S6K_f |
| MEK_f^1^ | (RAF_f \| MAP3K8) & !ERK_f | RAF_f \| MAP3K8 \| !ERK_f |
| PTEN | (PTEN_g \| ROCK1) & !(SRC \| CBPp300_c \| GSK3_f) | RTPK_f = (MMP_f \| RTPK_g) \|  !(MAPK14 \| MEK_f) |
| RAC_f | (DVL_f \| mTORC2_c \| VAV1 \| TIAM1) & !ARHGAP24 | DVL_f \| mTORC2_c \| VAV1 \| TIAM1 \| !ARHGAP24 |
| RAF_f | KRAS & !(AKT_f \| RHEB \| ERK_f) | KRAS \| !(AKT_f \| RHEB \| ERK_f) |
| REL_f | (CBPp300_c \| IKBKB \| MSK_f) & !STAT1 | CBPp300_c \| IKBKB \| MSK_f \| !STAT1 |
| SMAD3 | (TGFBR1 \| JNK_f \| MAPK14 \| ACVR1) & !(SMAD6 \| GSK3_f \| AKT_f \| ERK_f \| PPM1A \| SKI \| SMAD7) | TGFBR1 \| JNK_f \| MAPK14 \| ACVR1 \| !(SMAD6 \| GSK3_f \| AKT_f \| ERK_f \| PPM1A \| SKI \| SMAD7) |
| SMAD4 | (SMAD1 \| ERK_f \| SMAD5 \| SMAD2 \| PIAS1 \| SMAD3) & !(SMAD6 \| SKI \| SMURF1 \| SMAD7) | SMAD1 \| ERK_f \| SMAD5 \| SMAD2 \| PIAS1 \| SMAD3 \| !(SMAD6 \| SKI \| SMURF1 \| SMAD7) |
| SRC | RTPK_f & !CSK | RTPK_f \| !CSK |
| TGFBR1 | TGFBR2 & !(SMAD6 \| SMURF1 \| SMURF2 \| SMAD7) | TGFBR2 \| !(SMAD6 \| SMURF1 \| SMURF2 \| SMAD7) |
| TSC_f | GSK3_f & !(AKT_f \| ERK_f \| IKBKB \| RSK_f) | GSK3_f \| !(AKT_f \| ERK_f \| IKBKB \| RSK_f) |
| ^1^changed that a stable state can be reached | | |

Supplementary Table 12 – Synergy predictions for the AGS cell line using PKN informed with different activity profiles.

Abbreviations: true positive (TP), false negative (FN), true negative (TN), false positive (FP), positive predictive value (PPV), negative predictive value (NPV), Matthews’s correlation (MCC). Random - expected average count for respective values by chance when selecting the number of predicted synergistic combinations from the pool of possible drug combinations, ± standard error of the mean (n = 100,000).

| AGS | Literature | | Omics-inferred | | Combined | |
| --- | --- | --- | --- | --- | --- | --- |
|  | Prediction | Random  (average) | Prediction | Random  (average) | Prediction | Random  (average) |
| TP | 4 | 1.0 | 3 | 0.4 | 5 | 1.8 |
| FN | 10 | 13.0 | 12 | 14.6 | 10 | 13.3 |
| TN | 116 | 113.0 | 137 | 134.4 | 130 | 121.7 |
| FP | 6 | 9.0 | 1 | 3.6 | 5 | 15.3 |
| Sensitivity [%] | 28.57 | 7.3 | 20.0 | 2.6 | 33.3 | 11.2 |
| PPV [%] | 40.0 | 10.3 | 75.0 | 9.8 | 50.0 | 9.9 |
| NPV [%] | 92.1 | 89.7 | 91.9 | 90.2 | 92.9 | 90.1 |
| MCC | 0.28 | 0.00 | 0.36 | 0.00 | 0.36 | 0.00 |

Supplementary Table 13 - Synergy predictions for the SW-620 cell line using PKN informed with different activity profiles.

Abbreviations: true positive (TP), false negative (FN), true negative (TN), false positive (FP), positive predictive value (PPV), negative predictive value (NPV), Matthews’s correlation (MCC). Random - expected average count for respective values by chance when selecting the number of predicted synergistic combinations from the pool of possible drug combinations (n = 100,000).

| SW-620 | Literature | | Omics-inferred | | Combined | |
| --- | --- | --- | --- | --- | --- | --- |
|  | Prediction | Random  (average) | Prediction | Random  (average) | Prediction | Random  (average) |
| TP | 2 | 1.3 | 1 | 0.3 | 2 | 1.8 |
| FN | 8 | 8.7 | 9 | 9.7 | 8 | 7.2 |
| TN | 123 | 122.3 | 140 | 139.3 | 132 | 114.8 |
| FP | 17 | 17.7 | 3 | 3.7 | 11 | 28.2 |
| Sensitivity [%] | 20.0 | 12.7 | 10 | 2.6 | 20.0 | 19.8 |
| PPV [%] | 10.5 | 6.7 | 25.0 | 6.6 | 15.4 | 5.9 |
| NPV [%] | 94.0 | 93.3 | 94.0 | 93.5 | 94.3 | 94.1 |
| MCC | 0.06 | 0.00 | 0.12 | 0.00 | 0.11 | 0.00 |

Supplementary Table 14 - Synergy predictions for the AGS cell line using subset of combined baseline data.

Abbreviations: true positive (TP), false negative (FN), true negative (TN), false positive (FP), positive predictive value (PPV), negative predictive value (NPV), Matthews’s correlation (MCC). Random - expected average count for respective values by chance when selecting the number of predicted synergistic combinations from the pool of possible drug combinations, ± standard error of the mean (n = 100,000).

| AGS | Combined | | High-influence Nodes | | Low-influence Nodes | |
| --- | --- | --- | --- | --- | --- | --- |
|  | Prediction | Random  (average) | Prediction | Random  (average) | Prediction | Random  (average) |
| TP | 8 | 1.8 | 8 | 1.7 | 2 | 0.6 |
| FN | 7 | 13.3 | 7 | 13.3 | 13 | 14.4 |
| TN | 128 | 121.7 | 128 | 121.7 | 134 | 132.6 |
| FP | 9 | 15.3 | 9 | 15.3 | 4 | 5.4 |
| Sensitivity [%] | 53.3 | 11.2 | 53.3 | 11.2 | 13.3 | 3.9 |
| PPV [%] | 47.1 | 9.9 | 47.1 | 9.9 | 33.3 | 9.8 |
| NPV [%] | 94.8 | 90.1 | 94.8 | 90.1 | 91.2 | 90.2 |
| MCC | 0.44 | 0.00 | 0.44 | 0.00 | 0.16 | 0.00 |

Supplementary Table 15 – Synergy predictions for the SW-620 cell line using subset of combined baseline data.

Abbreviations: true positive (TP), false negative (FN), true negative (TN), false positive (FP), positive predictive value (PPV), negative predictive value (NPV), Matthews’s correlation (MCC). Random - expected average count for respective values by chance when selecting the number of predicted synergistic combinations from the pool of possible drug combinations, ± standard error of the mean (n = 100,000).

| SW-620 | Combined | | High-influence Nodes | | Low-influence Nodes | |
| --- | --- | --- | --- | --- | --- | --- |
|  | Prediction | Random  (average) | Prediction | Random  (average) | Prediction | Random  (average) |
| TP | 4 | 1.8 | 2 | 1.0 | 1 | 0.5 |
| FN | 5 | 7.2 | 7 | 8.0 | 9 | 9.5 |
| TN | 117 | 114.8 | 114 | 113.0 | 137 | 136.5 |
| FP | 26 | 28.2 | 13 | 14.0 | 6 | 6.5 |
| Sensitivity [%] | 44.4 | 19.8 | 22.2 | 11.0 | 10 | 4.6 |
| PPV [%] | 13.3 | 5.9 | 13.3 | 6.6 | 14.3 | 6.6 |
| NPV [%] | 95.9 | 94.1 | 94.2 | 93.4 | 93.8 | 93.5 |
| MCC | 0.16 | 0.00 | 0.10 | 0.00 | 0.07 | 0.00 |

Supplementary Table 16 - Synergy predictions for the DU-145 cell line using subset of combined baseline data.

Abbreviations: true positive (TP), false negative (FN), true negative (TN), false positive (FP), positive predictive value (PPV), negative predictive value (NPV), Matthews’s correlation (MCC). Random - expected average count for respective values by chance when selecting the number of predicted synergistic combinations from the pool of possible drug combinations (n = 100,000).

| DU-145 | Combined | | High-influence Nodes | | Low-influence Nodes | |
| --- | --- | --- | --- | --- | --- | --- |
|  | Prediction | Random  (average) | Prediction | Random  (average) | Prediction | Random  (average) |
| TP | 3 | 1.6 | 6 | 1.6 | 3 | 1.2 |
| FN | 14 | 15.4 | 10 | 14.4 | 14 | 15.8 |
| TN | 125 | 123.6 | 125 | 120.6 | 128 | 126.2 |
| FP | 11 | 12.4 | 9 | 13.4 | 8 | 9.8 |
| Sensitivity [%] | 17.6 | 9.1 | 37.5 | 10.0 | 17.6 | 7.2 |
| PPV [%] | 21.4 | 11.1 | 40.0 | 10.7 | 27.3 | 11.2 |
| NPV [%] | 90.0 | 88.9 | 92.59 | 89.3 | 90.1 | 88.9 |
| MCC | 0.10 | 0.00 | 0.32 | 0.00 | 0.14 | 0.00 |

Supplementary Table 17 - Synergy predictions for the COLO 205 cell line using subset of combined baseline data.

Abbreviations: true positive (TP), false negative (FN), true negative (TN), false positive (FP), positive predictive value (PPV), negative predictive value (NPV), Matthews’s correlation (MCC). Random - expected average count for respective values by chance when selecting the number of predicted synergistic combinations from the pool of possible drug combinations, ± standard error of the mean (n = 100,000).

| COLO 205 | Combined | | High-influence Nodes | | Low-influence Nodes | |
| --- | --- | --- | --- | --- | --- | --- |
|  | Prediction | Random  (average) | Prediction | Random  (average) | Prediction | Random  (average) |
| TP | 1 | 0.8 | 2 | 0.8 | 0 | 0.7 |
| FN | 7 | 7.2 | 5 | 6.2 | 7 | 6.3 |
| TN | 131 | 130.8 | 110 | 108.8 | 128 | 128.7 |
| FP | 14 | 14.2 | 12 | 13.2 | 15 | 14.3 |
| Sensitivity [%] | 12.5 | 9.8 | 28.57 | 10.9 | 0 | 10.0 |
| PPV [%] | 6.7 | 5.2 | 14.29 | 5.4 | 0 | 4.7 |
| NPV [%] | 94.9 | 94.8 | 95.65 | 94.6 | 94.8 | 95.3 |
| MCC | 0.02 | 0.00 | 0.14 | 0.00 | -0.07 | 0.00 |

Supplementary Table 18 - List of top 36 high-influence nodes per cell line ordered by number of affected combinations (high to low).

| AGS | COLO 205 | DU-145 | SW-620 |
| --- | --- | --- | --- |
| DKK_f | ITCH | PTEN | SMAD7 |
| DKK_g | JNK_f | RAF_f | MMP_f |
| LRP_f | PPP1CA | KRAS | KRAS |
| AXIN1 | SMAD7 | SHC1 | ILR_f |
| TCF7_f | SMURF2 | GRB2 | SOS1 |
| PTEN | RTPK_f | PTPN11 | PLCG1 |
| PPM1A | GSK3_f | GAB_f | SYK |
| PPP1CA | SHC1 | ILK | AKT_f |
| CHUK | RAF_f | PAK1 | MEK_f |
| ILK | PTEN | CHUK | PIK3CA |
| PAK1 | PTPN11 | TAB_f | RSK_f |
| SRC | KRAS | MAP3K7 | ERK_f |
| CTNNB1 | GAB_f | NLK | STAT1 |
| RTPK_f | GRB2 | ITCH | PIAS1 |
| RAF_f | MMP_f | SRC | MAPKAPK2 |
| KRAS | AKT_f | RTPK_f | RTPK_f |
| DUSP1 | DKK_f | MAPK14 | PPP1CA |
| ACVR1 | DKK_g | JNK_f | PDPK1 |
| SMURF2 | LRP_f | GSK3_f | RAF_f |
| BMPR2 | AXIN1 | MEK_f | STAT3 |
| SMAD7 | MEK_f | AKT_f | TRAF6 |
| MMP_f | RSK_f | PIK3CA | NLK |
| PDPK1 | ERK_f | TCF7_f | IRAK1 |
| GSK3_f | PDPK1 | CTNNB1 | SOCS1 |
| MEK_f | mTORC1_c | DKK_f | SOCS1_g |
| NLK | RHEB | DKK_g | ISGF3_c |
| AKT_f | TSC_f | LRP_f | TAB_f |
| RAC_f | AKT1S1 | AXIN1 | MAP3K7 |
| MYC | NLK | RSK_f | GSK3_f |
| ILR_f | SKP2 | mTORC1_c | mTORC1_c |
| IRAK1 | ILR_f | ISGF3_c | AKT1S1 |
| PIK3CA | IRAK1 | PPM1A | CTNNB1 |
| TRAF6 | DUSP1 | TRAF6 | AP1_c |
| TAB_f | MAPK14 | ERK_f | LEF1 |
| MAP3K7 | STAT3 | AKT1S1 | MAP3K8 |
| MAPK14 | TRAF6 | RHEB | mTORC2_c |

Supplementary Table 19 - Modified logical rules for high-influence AGS cell model.

| Node | Generic rule | Rule in high-influence AGS cell model |
| --- | --- | --- |
| PIP3 | PIK3CA & PDPK1 & !PTEN | PIK3CA & !PTEN |
| DKK_g | TCF7_f & !MYC | TCF7_f \| !MYC |
| AXIN1 | GSK3_f & !(LRP_f \| PPP1CA \| PPM1A) | GSK3_f \| !(LRP_f \| PPP1CA \| PPM1A) |
| RTPK_f | (MMP_f \| RTPK_g) & !(MAPK14 \| MEK_f) | MMP_f \| RTPK_g \| !(MAPK14 \| MEK_f) |
| RAF_f | KRAS & !(AKT_f \| RHEB \| ERK_f) | KRAS \| !(AKT_f \| RHEB \| ERK_f) |
| MEK_f | (RAF_f \| MAP3K8) & !ERK_f | RAF_f \| MAP3K8 \| !ERK_f |
| SOS1* | (GRB2 \| PLCG1) & !ERK_f | GRB2 \| PLCG1 \| !ERK_f |
| ERK_f* | MEK_f & !(PPP1CA \| DUSP6) | MEK_f \| !(PPP1CA \| DUSP6) |
| MAPK14 | MAP2K3 \| MAP2K4 \| !DUSP1 | (MAP2K3 \| MAP2K4) & !DUSP1 |
| LRP_f | (FZD_f \| MAPK14 \| JNK_f \| ERK_f) & !DKK_f | FZD_f \| MAPK14 \| JNK_f \| ERK_f \| !DKK_f |
| SRC | RTPK_f & !CSK | RTPK_f \| !CSK |
| DUSP1 | (MAPK14 \| DUSP1_g \| MSK_f) & !SKP2 | MAPK14 \| DUSP1_g \| MSK_f \| !SKP2 |
| RAC_f | (DVL_f \| mTORC2_c \| VAV1 \| TIAM1) & !ARHGAP24 | DVL_f \| mTORC2_c \| VAV1 \| TIAM1 \| !ARHGAP24 |
| SMAD7* | (EP300 \| SMAD7_g \| SMURF1) & !(AXIN1 \| SMURF2 \| ITCH) | (EP300 \| SMAD7_g \| SMURF1) \| !(AXIN1 \| SMURF2 \| ITCH) |
| IRAK1 | ILR_f & !SOCS1 | ILR_f \| !SOCS1 |
| TCF7_f | CTNNB1 & !NLK | CTNNB1 \| !NLK |
| *adjusted so that stable state can be reached | | |

Supplementary Table 20 - Modified rules in low-influence AGS cell model.

| Node | Generic rule | Rule in low-influence AGS cell model |
| --- | --- | --- |
| PIP3 | PIK3CA & PDPK1 & !PTEN | PIK3CA & !PTEN |
| MYC | (TCF7_f \| PLK1 \| STAT3) & !(GSK3_f \| MXD1) | (TCF7_f \| PLK1 \| STAT3) \| !(GSK3_f \| MXD1) |
| CFLAR | AKT_f & !ITCH | AKT_f \| !ITCH |
| AKT_f | (mTORC2_c \| ILK) & PIP3 & !PPP1CA | ((mTORC2_c \| ILK) & PIP3) \| !PPP1CA |
| RTPK_f * | (MMP_f \| RTPK_g) & !(MAPK14 \| MEK_f) | (MMP_f \| RTPK_g) \| !(MAPK14 \| MEK_f) |
| SMAD7* | (EP300 \| SMAD7_g \| SMURF1) & !(AXIN1 \| SMURF2 \| ITCH) | EP300 \| SMAD7_g \| SMURF1 \| !(AXIN1 \| SMURF2 \| ITCH) |
| SRC | RTPK_f & !CSK | RTPK_f \| !CSK |
| MDM2 | (AKT_f \| PPP1CA \| MDM2_g \| MAPKAPK2) & !S6K_f | (AKT_f \| PPP1CA \| MDM2_g \| MAPKAPK2) \| !S6K_f |
| BTRC | CK1_f & AXIN1 & GSK3_f & !LRP_f | (CK1_f & AXIN1 & GSK3_f) \| !LRP_f |
| DKK_g | TCF7_f & !MYC | TCF7_f \| !MYC |
| RAF_f | KRAS & !(AKT_f \| RHEB \| ERK_f) | KRAS \| !(AKT_f \| RHEB \| ERK_f) |
| LIMK2 | ROCK1 & !PRKCD | ROCK1 \| !PRKCD |
| SMAD5 | ACVR1 & !(SKI \| SMURF2) | ACVR1 \| !(SKI \| SMURF2) |
| TGFBR1 | TGFBR2 & !(SMAD6 \| SMURF1 \| SMURF2 \| SMAD7) | TGFBR2 \| !(SMAD6 \| SMURF1 \| SMURF2 \| SMAD7) |
| TGFBR2 | TGFB1 & !(SMURF1 \| SMURF2) | TGFB1 \| !(SMURF1 \| SMURF2) |
| JNK_f | (MAP2K4 \| MAP2K7 \| PAK1) & !DUSP1 | MAP2K4 \| MAP2K7 \| PAK1 \| !DUSP1 |
| DVL_f * | (FZD_f \| SMAD1) & !ITCH | FZD_f \| SMAD1 \| !ITCH |
| *adjusted so that stable state can be reached | | |

Supplementary Table 21 - Modified rules for high-influence DU-145 model.

| Node | Generic rule | Rule in high-influence DU-145 cell model |
| --- | --- | --- |
| PIP3 | PIK3CA & !PTEN | PIK3CA \| !PTEN |
| RAF_f | KRAS & !(AKT_f \| RHEB \| ERK_f) | KRAS \| !(AKT_f \| RHEB \| ERK_f) |
| GAB_f | GRB2 & !ERK_f | GRB2 \| !ERK_f |
| TAB_f | TRAF6 & !MAPK14 | TRAF6 \| !MAPK14 |
| SRC | RTPK_f & !CSK | RTPK_f \| !CSK |
| RTPK_f | (MMP_f \| RTPK_g) & !(MAPK14 \| MEK_f) | (MMP_f \| RTPK_g) \| !(MAPK14 \| MEK_f) |
| MEK_f | (RAF_f \| MAP3K8) & !ERK_f | (RAF_f \| MAP3K8) \| !ERK_f |
| LRP_f | (FZD_f \| MAPK14 \| JNK_f \| ERK_f) & !DKK_f | (FZD_f \| MAPK14 \| JNK_f \| ERK_f) \| !DKK_f |
| AXIN1 | GSK3_f & !(LRP_f \| PPP1CA \| PPM1A) | GSK3_f \| !(LRP_f \| PPP1CA \| PPM1A) |
| ERK_f | MEK_f & !(PPP1CA \| DUSP6) | MEK_f \| !(PPP1CA \| DUSP6) |
| RAC_f * | (DVL_f \| mTORC2_c \| VAV1 \| TIAM1) & !ARHGAP24 | (DVL_f \| mTORC2_c \| VAV1 \| TIAM1) \| !ARHGAP24 |
| CDC42* | SRC & !ARHGAP24 | SRC \| !ARHGAP24 |
| TCF7_f | CTNNB1 & !NLK | CTNNB1 \| !NLK |
| SMAD4* | (SMAD1 \| ERK_f \| SMAD5 \| SMAD2 \| PIAS1 \| SMAD3) & !(SMAD6 \| SKI \| SMURF1 \| SMAD7) | (SMAD1 \| ERK_f \| SMAD5 \| SMAD2 \| PIAS1 \| SMAD3) \| !(SMAD6 \| SKI \| SMURF1 \| SMAD7) |
| DKK_g | TCF7_f & !MYC | TCF7_f \| !MYC |
| *adjusted so that stable state can be reached | | |

Supplementary Table 22 - Modified rules for low-influence DU-145 model.

| Node | Generic rule | Rule in low-influence DU-145 cell model |
| --- | --- | --- |
| PIP3 | PIK3CA & !PTEN | PIK3CA \| !PTEN |
| SRC | RTPK_f & !CSK | RTPK_f \| !CSK |
| RTPK_f * | (MMP_f \| RTPK_g) & !(MAPK14 \| MEK_f) | (MMP_f \| RTPK_g) \| !(MAPK14 \| MEK_f) |
| SMAD5* | ACVR1 & !(SKI \| SMURF2) | ACVR1 \| !(SKI \| SMURF2) |
| CDC42* | SRC & !ARHGAP24 | SRC \| !ARHGAP24 |
| JNK_f * | (MAP2K4 \| MAP2K7 \| PAK1) & !DUSP1 | (MAP2K4 \| MAP2K7 \| PAK1) \| !DUSP1 |
| TAB_f * | TRAF6 & !MAPK14 | TRAF6 \| !MAPK14 |
| SMAD4* | (SMAD1 \| ERK_f \| SMAD5 \| SMAD2 \| PIAS1 \| SMAD3) & !(SMAD6 \| SKI \| SMURF1 \| SMAD7) | (SMAD1 \| ERK_f \| SMAD5 \| SMAD2 \| PIAS1 \| SMAD3) \| !(SMAD6 \| SKI \| SMURF1 \| SMAD7) |
| MDM2 | (AKT_f \| PPP1CA \| MDM2_g \| MAPKAPK2) & !S6K_f | (AKT_f \| PPP1CA \| MDM2_g \| MAPKAPK2) \| !S6K_f |
| CFLAR | AKT_f & !ITCH | AKT_f \| !ITCH |
| *adjusted so that stable state can be reached | | |

Supplementary Table 23 - Modified rules for high-influence SW-620 model.

| Node | Generic rule | Rule in high-influence SW-620 cell model |
| --- | --- | --- |
| PIP3 | PIK3CA & !PTEN | PIK3CA \| !PTEN |
| GAB_f | GRB2 & !ERK_f | GRB2 \| !ERK_f |
| AKT_f | (mTORC2_c \| ILK) & PIP3 & !PPP1CA | (mTORC2_c \| ILK) & PIP3 \| !PPP1CA |
| MEK_f | (RAF_f \| MAP3K8) & !ERK_f | (RAF_f \| MAP3K8) \| !ERK_f |
| ERK_f | MEK_f & !(PPP1CA \| DUSP6) | MEK_f \| !(PPP1CA \| DUSP6) |
| RTPK_f | (MMP_f \| RTPK_g) & !(MAPK14 \| MEK_f) | MMP_f \| RTPK_g \| !(MAPK14 \| MEK_f) |
| PPP1CA | SMAD7 & !RTPK_f | SMAD7 \| !RTPK_f |
| RAF_f | KRAS & !(AKT_f \| RHEB \| ERK_f) | KRAS \| !(AKT_f \| RHEB \| ERK_f) |
| SOS1* | (GRB2 \| PLCG1) & !ERK_f | GRB2 \| PLCG1 \| !ERK_f |
| MAPK14 | MAP2K3 \| MAP2K4 \| !DUSP1 | (MAP2K3 \| MAP2K4) & !DUSP1 |
| SMAD7 | (EP300 \| SMAD7_g \| SMURF1) & !(AXIN1 \| SMURF2 \| ITCH) | (EP300 \| SMAD7_g \| SMURF1) \| !(AXIN1 \| SMURF2 \| ITCH) |
| TAB_f | TRAF6 & !MAPK14 | TRAF6 \| !MAPK14 |
| *adjusted so that stable state can be reached | | |

Supplementary Table 24 - Modified rules for low-influence SW-620 model.

| Node | Generic rule | Rule in low-influence SW-620 cell model |
| --- | --- | --- |
| PIP3 | PIK3CA & !PTEN | PIK3CA \| !PTEN |
| SRC | RTPK_f & !CSK | RTPK_f \| !CSK |
| RTPK_f | (MMP_f \| RTPK_g) & !(MAPK14 \| MEK_f) | (MMP_f \| RTPK_g) \| !(MAPK14 \| MEK_f) |
| SMAD1* | ACVR1 & !(GSK3_f \| ERK_f \| PPM1A \| SKI \| SMURF1 \| SMAD6) | ACVR1 \| !(GSK3_f \| ERK_f \| PPM1A \| SKI \| SMURF1 \| SMAD6) |
| SMAD7* | (EP300 \| SMAD7_g \| SMURF1) & !(AXIN1 \| SMURF2 \| ITCH) | (EP300 \| SMAD7_g \| SMURF1) \| !(AXIN1 \| SMURF2 \| ITCH) |
| TGFBR1 | TGFBR2 & !(SMAD6 \| SMURF1 \| SMURF2 \| SMAD7) | TGFBR2 \| !(SMAD6 \| SMURF1 \| SMURF2 \| SMAD7) |
| TGFBR2 | TGFB1 & !(SMURF1 \| SMURF2) | TGFB1 \| !(SMURF1 \| SMURF2) |
| MDM2 | (AKT_f \| PPP1CA \| MDM2_g \| MAPKAPK2) & !S6K_f | (AKT_f \| PPP1CA \| MDM2_g \| MAPKAPK2) \| !S6K_f |
| *adjusted so that stable state can be reached | | |

Supplementary Table 25 - Modified logical rules for high-influence COLO 205 model.

| Node | Generic rule | Rule in high-influence COLO 205 cell model |
| --- | --- | --- |
| RAF_f | KRAS & !(AKT_f \| RHEB \| ERK_f) | KRAS \| !(AKT_f \| RHEB \| ERK_f) |
| AXIN1 | GSK3_f & !(LRP_f \| PPP1CA \| PPM1A) | GSK3_f \| !(LRP_f \| PPP1CA \| PPM1A) |
| ERK_f | MEK_f & !(PPP1CA \| DUSP6) | MEK_f \| !(PPP1CA \| DUSP6) |
| MEK_f * | (RAF_f \| MAP3K8) & !ERK_f | (RAF_f \| MAP3K8) \| !ERK_f |
| IKBKB* | MAP3K7 & !(TP53 \| PLK1 \| PPM1A) | MAP3K7 \| !(TP53 \| PLK1 \| PPM1A) |
| TAB_f * | TRAF6 & !MAPK14 | TRAF6 \| !MAPK14 |
| TGFBR2* | TGFB1 & !(SMURF1 \| SMURF2) | TGFB1 \| !(SMURF1 \| SMURF2) |
| SRC | RTPK_f & !CSK | RTPK_f \| !CSK |
| AKT_f | (mTORC2_c \| ILK) & PIP3 & !PPP1CA | (mTORC2_c \| ILK) & PIP3 \| !PPP1CA |
| MAPK14 | (MAP2K3 \| MAP2K4) \| !DUSP1 | (MAP2K3 \| MAP2K4) & !DUSP1 |
| RTPK_f | (MMP_f \| RTPK_g) & !(MAPK14 \| MEK_f) | (MMP_f \| RTPK_g) \| !(MAPK14 \| MEK_f) |
| TSC_f | GSK3_f & !(AKT_f \| ERK_f \| IKBKB \| RSK_f) | GSK3_f \| !(AKT_f \| ERK_f \| IKBKB \| RSK_f) |
| SMAD_7* | (EP300 \| SMAD7_g \| SMURF1) & !(AXIN1 \| SMURF2 \| ITCH) | (EP300 \| SMAD7_g \| SMURF1) \| !(AXIN1 \| SMURF2 \| ITCH) |
| *adjusted so that stable state can be reached | | |

Supplementary Table 26 - Modified logical rules for low-influence COLO 205 model.

| Node | Generic rule | Rule in low-influence COLO 205 cell model |
| --- | --- | --- |
| RTPK_f | (MMP_f \| RTPK_g) & !(MAPK14 \| MEK_f) | (MMP_f \| RTPK_g) \| !(MAPK14 \| MEK_f) |
| MEK_f | (RAF_f \| MAP3K8) & !ERK_f | (RAF_f \| MAP3K8) \| !ERK_f |
| ERK_f | MEK_f & !(PPP1CA \| DUSP6) | MEK_f \| !(PPP1CA \| DUSP6) |
| SOS1 | (GRB2 \| PLCG1) & !ERK_f | (GRB2 \| PLCG1) \| !ERK_f |
| SRC | RTPK_f & !CSK | RTPK_f \| !CSK |
| RAF_f * | KRAS & !(AKT_f \| RHEB \| ERK_f) | KRAS \| !(AKT_f \| RHEB \| ERK_f) |
| mTORC_2 * | (PIK3CA \| TSC_f) & !S6K_f | (PIK3CA \| TSC_f) \| !S6K_f |
| TGFBR1* | TGFBR2 & !(SMAD6 \| SMURF1 \| SMURF2 \| SMAD7) | TGFBR2 \| !(SMAD6 \| SMURF1 \| SMURF2 \| SMAD7) |
| SMAD7* | (EP300 \| SMAD7_g \| SMURF1) & !(AXIN1 \| SMURF2 \| ITCH) | (EP300 \| SMAD7_g \| SMURF1) \| !(AXIN1 \| SMURF2 \| ITCH) |
| LIMK2 | ROCK1 & !PRKCD | ROCK1 \| !PRKCD |
| SMAD5 | ACVR1 & !(SKI \| SMURF2) | ACVR1 \| !(SKI \| SMURF2) |
| CDC42 | SRC & !ARHGAP24 | SRC \| !ARHGAP24 |
| TGFBR2 | TGFB1 & !(SMURF1 \| SMURF2) | TGFB1 \| !(SMURF1 \| SMURF2) |
| DUSP1 | (MAPK14 \| DUSP1_g \| MSK_f) & !SKP2 | (MAPK14 \| DUSP1_g \| MSK_f) \| !SKP2 |
| REL_f | (CBPp300_c \| IKBKB \| MSK_f) & !STAT1 | (CBPp300_c \| IKBKB \| MSK_f) \| !STAT1 |
| BTRC | CK1_f & AXIN1 & GSK3_f & !LRP_f | CK1_f & AXIN1 & GSK3_f \| !LRP_f |
| JNK_f | (MAP2K4 \| MAP2K7 \| PAK1) & !DUSP1 | (MAP2K4 \| MAP2K7 \| PAK1) \| !DUSP1 |
| CFLAR | AKT_f & !ITCH | AKT_f \| !ITCH |
| BAX | (AKT_f \| PPP1CA \| MDM2_g \| MAPKAPK2) & !S6K_f | (AKT_f \| PPP1CA \| MDM2_g \| MAPKAPK2) \| !S6K_f |
| TAB_f | TRAF6 & !MAPK14 | TRAF6 \| !MAPK14 |
| *adjusted so that stable state can be reached | | |

Supplementary Table 27 - Model predictions using different HSA synergy cut-offs for each cell line.

| Cell line | HSA cut-off for synergy call | TP | FN | TN | FP | Sensitivity | Specificy | PPV | NPV | MCC |
| --- | --- | --- | --- | --- | --- | --- | --- | --- | --- | --- |
| AGS | ≤ -0.08 | 8 | 18 | 117 | 9 | 30,8 % | 92,9 % | 47,1 % | 86,7 % | 0,28 |
|  | ≤ -0.11 | 8 | 7 | 128 | 9 | 53,3 % | 93,4 % | 47,1 % | 94,8 % | 0,44 |
|  | ≤ -0.13 | 5 | 2 | 133 | 12 | 71,4 % | 91,7 % | 29,4 % | 98,5 % | 0,42 |
| SW-620 | ≤ -0.08 | 12 | 8 | 114 | 18 | 60,0 % | 86,4 % | 40,0 % | 93,4 % | 0,39 |
|  | ≤ -0.11 | 4 | 5 | 117 | 26 | 44,4 % | 81,8 % | 13,3 % | 95,9 % | 0,16 |
|  | ≤ -0.13 | 4 | 3 | 119 | 26 | 57,1 % | 82,1 % | 13,3 % | 97,5 % | 0,21 |
| COLO 205 | ≤ -0.08 | 3 | 12 | 126 | 12 | 20,0 % | 91,3 % | 20,0 % | 91,3 % | 0,11 |
|  | ≤ -0.11 | 1 | 7 | 131 | 14 | 12,5 % | 90,3 % | 6,7 % | 94,9 % | 0,02 |
|  | ≤ -0.13 | 0 | 4 | 134 | 15 | 0,0 % | 89,9 % | 0,0 % | 97,1 % | 0,05 |
| DU-145 | ≤ -0.08 | 4 | 25 | 114 | 10 | 13,8 % | 91,9 % | 28,6 % | 82,0 % | 0,08 |
|  | ≤ -0.11 | 3 | 14 | 125 | 11 | 17,6 % | 91,9 % | 21,4 % | 89,9 % | 0,10 |
|  | ≤ -0.13 | 3 | 5 | 134 | 11 | 37,5 % | 92,4 % | 21,4 % | 96,4 % | 0,23 |
